# Genetically Predicted Sarcopenia Traits and the Risk of Barrett's Esophagus

**DOI:** 10.1002/fsn3.71148

**Published:** 2025-11-13

**Authors:** Jianfeng Zhou, Dexiu E, Yuwen Tan, Yixin Liu, Pinhao Fang, Zhi Ye, Yushang Yang

**Affiliations:** ^1^ Department of Thoracic Surgery, Med+X Center for Informatics, West China Hospital Sichuan University Chengdu Sichuan China; ^2^ Department of Thoracic Surgery Qinghai Provincial People's Hospital Xining Qinghai China; ^3^ Guangdong Provincial Engineering Research Center of Molecular Imaging, the Fifth Affiliated Hospital Sun Yat‐Sen University Zhuhai Guangdong China

**Keywords:** appendicular lean mass, Barrett's esophagus, causal association, grip strength, Mendelian randomization, walking speed

## Abstract

A growing body of cohort research suggests a link between sarcopenia and Barrett's esophagus (BE); however, a definitive causal connection has yet to be established. Consequently, this study aims to evaluate the potential causal association between sarcopenia and BE. We obtained data on single‐nucleotide polymorphisms (SNPs) linked to appendicular lean mass, hand grip strength, and walking speed, as well as outcome data related to Barrett's esophagus, from publicly available genome‐wide association studies (GWAS). Utilizing these datasets, we conducted a multivariable Mendelian randomization (MR) analysis to explore potential causal associations. Univariable MR revealed significant inverse associations between sarcopenia‐related traits and BE risk. Specifically, higher appendicular lean mass, stronger grip strength, and faster walking pace were each associated with reduced BE risk (all *p* < 0.05). Bidirectional MR indicated no reverse causality from BE to sarcopenia. In comprehensive multivariable MR models simultaneously adjusting for BMI, smoking, alcohol intake, dietary macronutrients, vitamin D, and calcium, appendicular lean mass (OR = 0.681, *p* = 0.028) and walking pace (OR = 0.113, *p* = 0.019) remained significantly protective against BE, whereas the associations for hand grip strength were attenuated and became non‐significant. Sensitivity analyses confirmed the absence of horizontal pleiotropy and showed consistent findings across MR methods. The multivariable MR analysis demonstrated a positive causal link between sarcopenia and BE, even after adjusting for confounders. However, as all GWAS datasets were from European populations, the generalizability of these findings requires further validation. Large‐scale prospective studies are needed to confirm these results and evaluate whether muscle‐targeted strategies can help manage this condition.

## Graphical abstract

This Mendelian randomization study investigated the causal association between sarcopenia‐related traits and Barrett's esophagus (BE). Higher appendicular lean mass and faster walking pace were robustly associated with reduced BE risk, even after adjusting for BMI, smoking, alcohol intake, dietary factors, vitamin D, and calcium. These findings suggest that muscle‐targeted strategies may represent novel preventive approaches against BE.

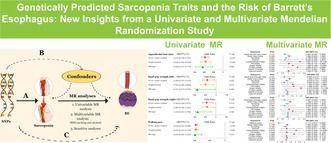

## 1 Introduction

Barrett's esophagus (BE) is a well‐recognized premalignant condition in which the normal stratified squamous epithelium of the distal esophagus is replaced by metaplastic columnar epithelium, often as a consequence of chronic gastroesophageal reflux disease (GERD) (Bresalier 2009; Eluri and Shaheen 2017). The development of BE represents a critical step in the progression toward esophageal adenocarcinoma (EAC), a malignancy with a dismal prognosis and rapidly increasing incidence in Western and now also Asian populations (Sharma 2022). Epidemiological studies have estimated the prevalence of BE to range from 1% to 2% in the general adult population, with higher rates observed in males, individuals over 50 years of age, and those with longstanding GERD symptoms. The exact pathogenesis of BE remains incompletely understood, but it is believed to involve repeated exposure of the esophageal mucosa to acidic and bile‐containing refluxate, leading to chronic inflammation, epithelial injury, and metaplastic transformation (Que et al. 2019; Schlottmann et al. 2018). In addition to reflux, several modifiable and non‐modifiable risk factors—such as obesity, smoking, alcohol consumption, and genetic susceptibility—have been implicated in BE development. Notably, visceral adiposity and metabolic dysfunction have been shown to promote systemic and local pro‐inflammatory environments that may facilitate the metaplastic process in the esophageal lining (Stawinski et al. 2023). Despite advances in endoscopic surveillance and ablative therapy, the overall progression rate from BE to high‐grade dysplasia or adenocarcinoma remains a significant concern, emphasizing the need for better identification of upstream risk factors and preventive strategies.

Sarcopenia, a progressive decline in skeletal muscle mass and strength, has traditionally been associated with aging but is increasingly recognized in various chronic disease states and metabolic disorders (Khaddour et al. 2020). Clinically, sarcopenia contributes to physical frailty, reduced mobility, impaired quality of life, and increased risk of morbidity and mortality. Recent shifts in research paradigms have led to the appreciation of sarcopenia as not only a musculoskeletal condition but also a systemic syndrome with widespread physiological effects. (Peterson and Mozer 2017) Of particular interest is its emerging connection with gastrointestinal disorders, including liver disease, colorectal cancer, and functional bowel syndromes. Several lines of evidence suggest that sarcopenia may influence gastrointestinal health through mechanisms such as impaired visceral motility, reduced abdominal wall strength, alterations in gut microbiota, and chronic low‐grade inflammation. In the context of BE, sarcopenia may promote or exacerbate pathological reflux through diminished esophageal clearance and diaphragmatic support, increased intra‐abdominal pressure, and metabolic disturbances associated with altered body composition, especially when coexisting with visceral obesity. Observational cohort studies have reported higher rates of GERD and BE in individuals with reduced muscle strength or mass, but these associations remain correlative and subject to residual confounding. (Kim et al. 2019; Imagama et al. 2019) Given the shared risk profile between sarcopenia and BE—particularly in aging populations—it is crucial to further explore whether sarcopenia plays an active role in the pathogenesis of BE or simply serves as a surrogate marker of overall frailty and metabolic imbalance.

The lack of rigorous randomized controlled trials specifically addressing this association complicates the establishment of clear causality. Mendelian randomization (MR) analysis offers a robust method to investigate causal relationships distinct from traditional observational studies. The inherent allelic randomization in MR ensures that genetic predispositions occur before the development of BE, helping to prevent reverse causation bias. Furthermore, the random segregation and independent assortment of genetic polymorphisms at conception allow for the use of genetic markers as instrumental variables (IVs) for sarcopenia, thereby controlling for confounding factors. (Davey Smith and Ebrahim 2005; Maier et al. 2018) The extensive availability of genome‐wide association studies (GWASs) enhances the capacity of researchers to assess causality rigorously. Consequently, MR analysis provides a valuable tool to answer critical questions about the relationship between sarcopenia and BE, whether it is negative, neutral, or positive.

## 2 Methods

### 2.1 Study Design

**FIGURE 1:**
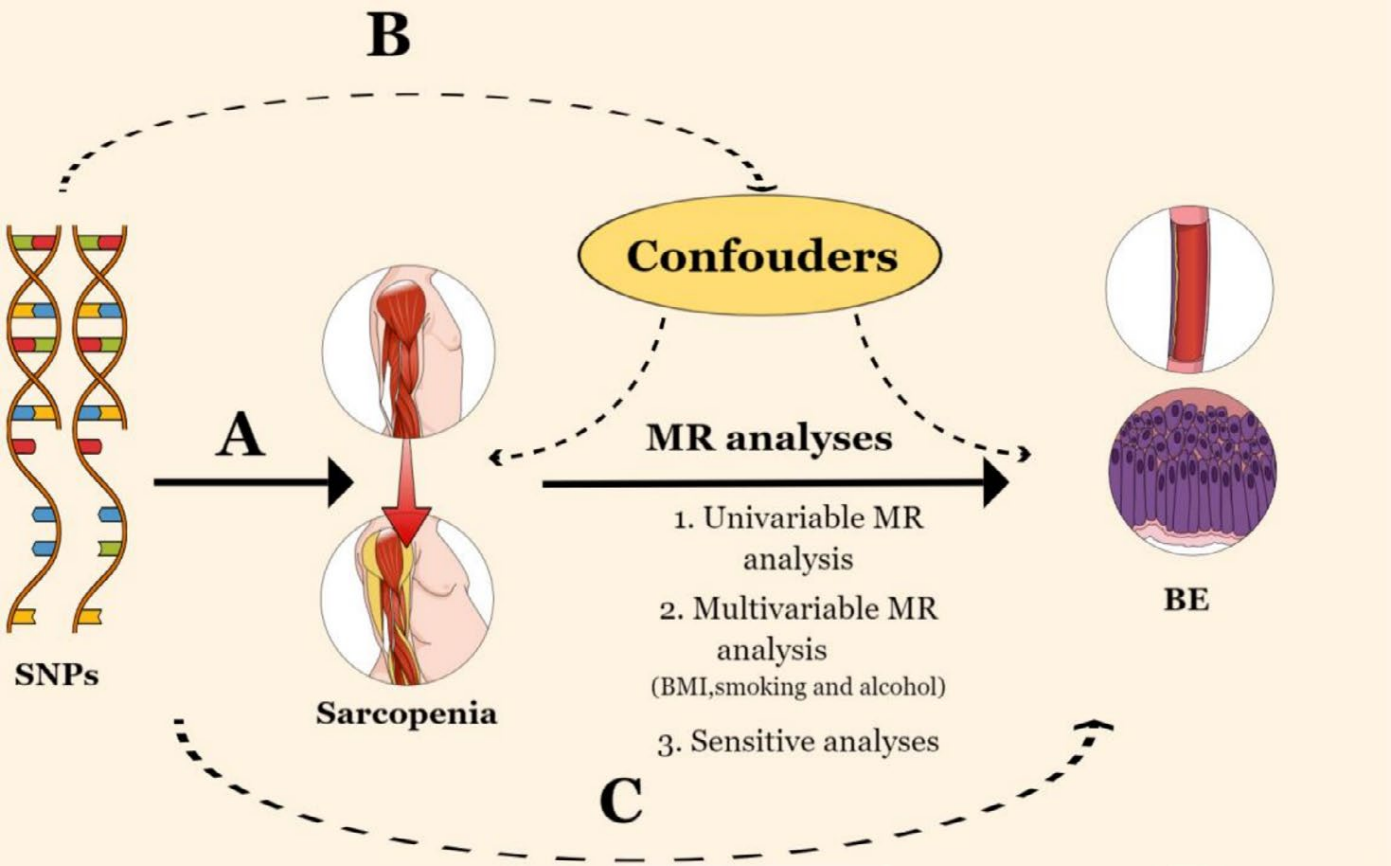
Three key assumptions of the Mendelian randomization study: (A) SNPs are strongly associated with physical performance or parameters of sarcopenia; (B) SNPs are independent of confounders; (C) SNPs must only affect BE via sarcopenia. BE, Barrett's esophagus; MR, Mendelian randomization; SNP, Single‐nucleotide polymorphism.

Through the use of both univariable and multivariable MR analyses, our study evaluated the causal effect of sarcopenia on the development of BE. Figure 1 outlines the study design and highlights the three core assumptions of MR: (A) a strong association between single nucleotide polymorphisms (SNPs) and sarcopenia, (B) independence of SNPs from known confounders, and (C) the effect of SNPs on BE is mediated exclusively through sarcopenia (Emdin et al. 2017).

### 2.2 Data Sources

This study employed publicly available GWAS summary‐level data, primarily derived from European populations, including both male and female participants. According to the European Working Group on Sarcopenia in Older People (EWGSOP) (Frisoli Junior et al. 2025), sarcopenia is diagnosed based on the presence of low muscle mass, low muscle strength, and/or impaired physical performance. In this study, we used three representative and GWAS‐available sarcopenia‐related traits—appendicular lean mass, hand grip strength, and walking pace—as proxies to explore their potential causal effects on Barrett's esophagus. Specifically, appendicular lean mass data (*n* = 450,243) were sourced from the GWAS dataset ebi‐a‐GCST90000025, published in 2020, comprising 18,071,518 SNPs. Hand grip strength (left and right) data (*n* = 461,026 and 461,089, respectively) originated from the UK Biobank via the MRC Integrative Epidemiology Unit (GWAS IDs: ukb‐b‐7478 and ukb‐b‐10,215, respectively), both conducted in 2018 and encompassing 9,851,867 SNPs. Walking pace (*n* = 459,915) was extracted from ukb‐b‐4711, also under MRC‐IEU, with 9,851,867 SNPs. BE outcome data were obtained from ebi‐a‐GCST90000515 (*n* = 56,429; 13,358 cases and 43,071 controls), published in 2021 with 2,320,520 SNPs.

To avoid potential bias from sample overlap, we confirmed that the GWAS datasets used for exposures (appendicular lean mass, hand grip strength, walking pace) and outcomes (Barrett's esophagus) were derived from non‐overlapping, independent cohorts. The exposure GWAS datasets were primarily obtained from UK Biobank or IEU Open GWAS sources distinct from the BE outcome datasets, ensuring the validity of the two‐sample MR design (Table 1). Furthermore, a multivariable MR analysis was conducted to explore the potential mediating effects of BMI, smoking, alcohol consumption, protein intake, lipid intake, carbohydrate intake, sugar intake, as well as vitamin D and calcium levels—key risk factors for BE that have also been linked to sarcopenia. For confounder adjustment in multivariable MR, we used: BMI from ieu‐b‐4815, a within‐family GWAS of 51,852 individuals (2022); Smoking from ebi‐a‐GCST009967 (*n* = 4676, 2020); Alcohol consumption from ieu‐b‐4834 (*n* = 83,626, 2022), derived from the MRC‐IEU. Summary statistics for protein intake, lipid intake, carbohydrate intake, and sugar intake were derived from a large‐scale GWAS of 268,922 European individuals conducted by Meddens et al. (2021), as summarized in Xiang et al. (2025). These statistics were based on energy‐adjusted dietary intake data from food‐frequency questionnaires and 24‐h dietary recalls. Participants with macronutrient‐ or calorie‐restricted diets were excluded to minimize dietary confounding (Xiang et al. 2025), as well as vitamin D and calcium levels obtained from ebi‐a‐GCST005367 (*n* = 79,366) and ebi‐a‐GCST90025990 (*n* = 400,792), which were published in 2018 with 2,538,249 SNPs and 2021 with 4,218,949 SNPs, respectively.

**TABLE 1:** The characteristics of GWAS data.

Trait	Sample size	*n* case	n control	GWAS ID	Consortium	Year	Population	*n* SNP
Appendicular lean mass	450,243	NA	NA	ebi‐a‐GCST90000025	NA	2020	European	18,071,518
Hand grip strength (left)	461,026	NA	NA	ukb‐b‐7478	MRC‐IEU	2018	European	9,851,867
Hand grip strength (right)	461,089	NA	NA	ukb‐b‐10,215	MRC‐IEU	2018	European	9,851,867
Walking pace	459,915	NA	NA	ukb‐b‐4711	MRC‐IEU	2018	European	9,851,867
BE	56,429	13,358	43,071	ebi‐a‐GCST90000515	NA	2021	European	2,320,520
BMI	51,852	NA	NA	ieu‐b‐4815	Within family GWAS consortium	2022	European	NA
Smoking behavior (cigarette pack‐years)	4676	NA	NA	ebi‐a‐GCST009967	NA	2020	European	8,649,675
Alcoholic drinks per week	335,394	NA	NA	ieu‐b‐73	GWAS and Sequencing Consortium of Alcohol and Nicotine use	2019	European	11,887,865
Protein intake (energy‐adjusted)	268,922	NA	NA	Meddens et al. (PMID: 34040186)	NA	2021	European	NA
Lipid intake (energy‐adjusted)	268,922	NA	NA	Meddens et al. (PMID: 34040186)	NA	2021	European	NA
Carbohydrate intake (energy‐adjusted)	268,922	NA	NA	Meddens et al. (PMID: 34040186)	NA	2021	European	NA
Sugar intake (energy‐adjusted)	268,922	NA	NA	Meddens et al. (PMID: 34040186)	NA	2021	European	NA
Vitamin D levels	79,366	NA	NA	ebi‐a‐GCST005367	NA	2018	European	2,538,249
Calcium levels	400,792	NA	NA	ebi‐a‐GCST90025990	NA	2021	European	4,218,949

Abbreviations: BE, Barrett's esophagus; BMI, body mass index; GWAS, genome‐wide association studies; SNP, single‐nucleotide polymorphism.

Comprehensive details regarding the included studies are presented in Table 1. Since all GWAS data utilized in this analysis were publicly available and had received prior approval from the respective ethical review boards, no additional ethics approval was required for this study.

### 2.3 Selection and Validation of SNPs

To ensure the selection of relevant SNPs for analysis, three criteria were employed. First, SNPs linked to appendicular lean mass, hand grip strength, and walking pace were identified based on genome‐wide significance, using a threshold of *p* < 5 × 10^−8^. Second, the independence of selected SNPs was evaluated through pairwise linkage disequilibrium; (Machiela and Chanock 2015) SNPs with an *r* ^2^ > 0.001 within a clumping window of 10,000 kb were excluded if they were highly correlated with others or had a higher *p*‐value. Third, the F‐statistic was calculated to assess the strength of individual SNPs. Those with *F*‐statistics greater than 10 were considered robust enough to mitigate potential bias. (Feng et al. 2022) Simultaneously, SNPs with pleiotropic associations involving secondary traits, which could potentially introduce confounding into the causal estimation, were eliminated. This was accomplished using PhenoScanner v2 [PhenoScanner (http://cam.ac.uk), *p* < 1 × 10^−5^], ensuring adherence to the second assumption of SNP independence. Before conducting the MR analysis, data harmonization was carried out using the TwoSampleMR R package, which aligned the effect alleles of each SNP across exposure and outcome datasets, corrected strand inconsistencies, and excluded palindromic SNPs with intermediate allele frequencies to avoid ambiguity (Hemani et al. 2018; Bowden et al. 2017).

### 2.4 MR Analysis

The primary analysis employed an inverse‐variance weighted (IVW) meta‐analysis, applying a fixed‐effects model for univariable MR analysis and a random‐effects model for multivariable MR analysis. Since smoking traits, BMI, and alcohol consumption are potential risk factors for BE, multivariable MR analyses were conducted to adjust for these confounders and assess the direct impact of sarcopenia on BE. Two complementary MVMR strategies were applied: (Bresalier 2009) Stepwise MVMR sensitivity analyses—Each sarcopenia‐related trait (appendicular lean mass, grip strength, or walking pace) was modeled together with one potential confounder at a time (body mass index, smoking, alcohol consumption, dietary macronutrients, vitamin D, or calcium). This approach was designed to evaluate the stability of causal estimates under different adjustment scenarios. (Eluri and Shaheen 2017) Comprehensive joint MVMR—All potential confounders (BMI, smoking, alcohol, protein, fat, carbohydrate, sugar intake, vitamin D, and calcium) were included simultaneously to estimate the direct causal effect of each sarcopenia trait on BE risk.

The MR‐Egger intercept test, along with scatter plots and funnel plots, which are instrumental in detecting horizontal pleiotropy, was used to assess the potential influence of pleiotropic effects. Horizontal pleiotropy is suggested when the MR‐Egger intercept significantly deviates from zero (*p* < 0.05) or when asymmetry is observed in the funnel plot. (Bowden et al. 2015) To assess heterogeneity among the instrumental variables (IVs), we employed Cochrane's Q statistic, considering heterogeneity significant when the *p*‐value was less than 0.05. A leave‐one‐out sensitivity test was also performed to evaluate the influence of individual SNPs on the overall estimates. The robustness of the IVs in the multivariable MR analyses was determined by calculating the *F* statistic with the ‘MVMR’ package in R, with an F statistic exceeding 10 indicating strong instruments (Sanderson et al. 2021). All statistical analyses were executed using the “TwoSampleMR” and “MendelianRandomization” packages in R version 4.0.3, as provided by the R Foundation for Statistical Computing in Vienna, Austria.

## 3 Results

### 3.1 SNP Selection and Validation

In conclusion, the studies incorporated in our analysis were published between 2018 and 2022, predominantly involving European populations (Table 1). The instrumental variables selected for appendicular lean mass, left‐hand grip strength, right‐hand grip strength, and walking pace all reached genome‐wide significance, with *F*‐statistics exceeding 10 in the univariable MR analyses (Tables S1–S5), confirming their robustness.

### 3.2 Causal Association of Sarcopenia and BE via Univariable MR

**FIGURE 2:**
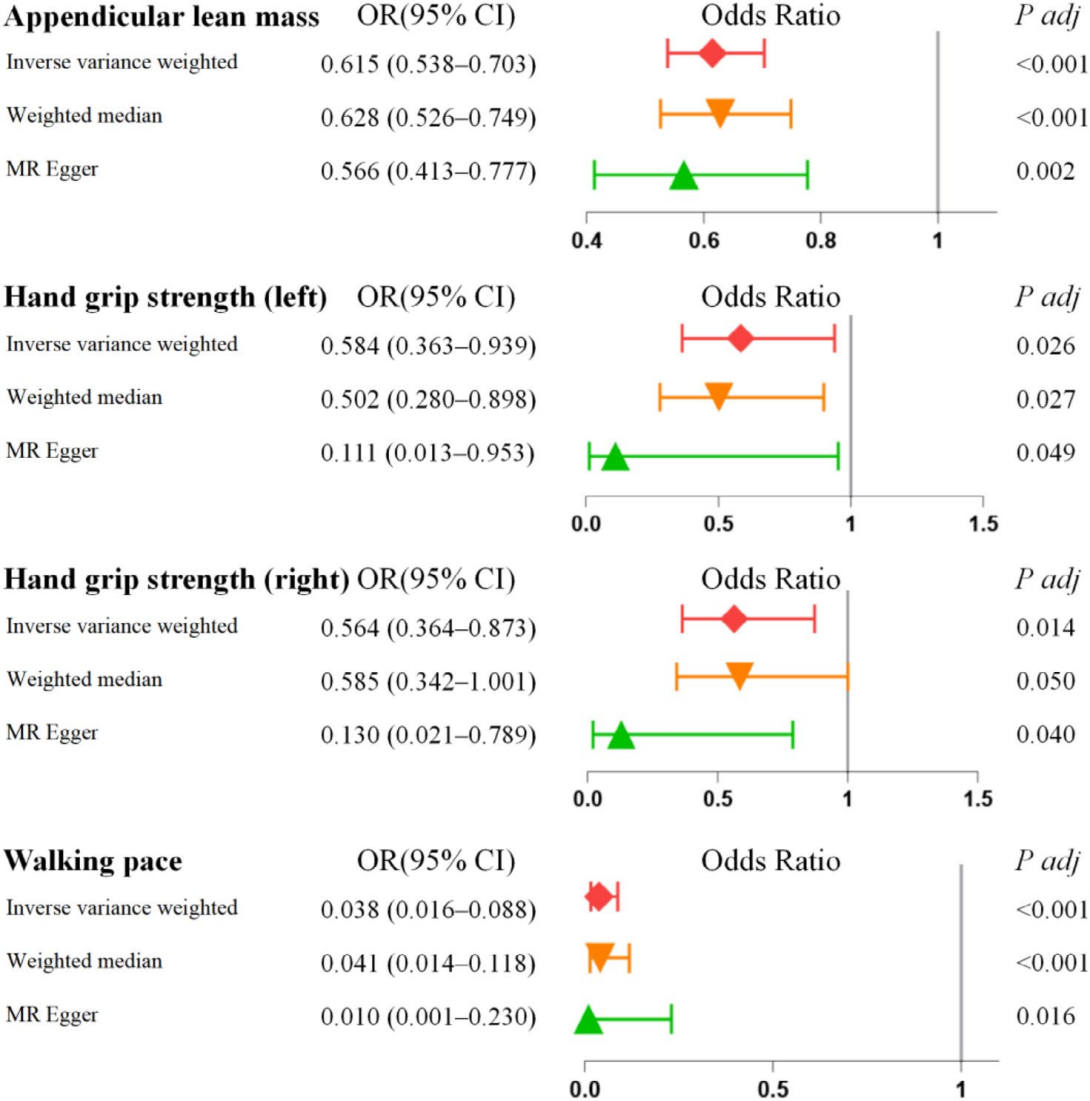
Associations of genetically predicted physical performance or parameters of sarcopenia with BE. The reported values were calculated by the fixed effects IVW method. BE, Barrett's esophagus; CI, confidence interval; IVW, inverse variance weighted method; OR, Odds ratio.

Using the IVW method in our primary MR model, we observed a significant relationship between appendicular lean mass and BE risk (OR = 0.615; 95% CI = 0.538–0.703; *p* < 0.001, Figure 2). Similarly, the associations for hand grip strength and walking pace were statistically significant, mirroring the findings for appendicular lean mass. Specifically, the left‐hand grip strength yielded an OR of 0.584 (95% CI = 0.363–0.939; *p* = 0.026), the right‐hand grip strength an OR of 0.564 (95% CI = 0.364–0.873; *p* = 0.014), and walking pace an OR of 0.038 (95% CI = 0.016–0.088; *p* < 0.001) (Figure 2).

### 3.3 Causal Association of BE and Sarcopenia‐Related Traits via Bidirectional MR

In the bidirectional MR analysis, no significant causal effect of BE on sarcopenia‐related traits was observed. Using the IVW method, the OR for the association between BE and sarcopenia traits was as follows: Appendicular lean mass: OR = 0.987 (95% CI: 0.971–1.003), *p* = 0.329; Hand grip strength (left): OR = 0.997 (95% CI: 0.986–1.008), *p* = 0.539; Hand grip strength (right): OR = 0.991 (95% CI: 0.977–1.005), *p* = 0.329; Walking pace: OR = 0.993 (95% CI: 0.981–1.005), *p* = 0.329. All results were non‐significant, suggesting no causal effect of BE on sarcopenia‐related traits (Figure S1).

### 3.4 Causal Association of Sarcopenia and BE via Multivariable MR

**FIGURE 3:**
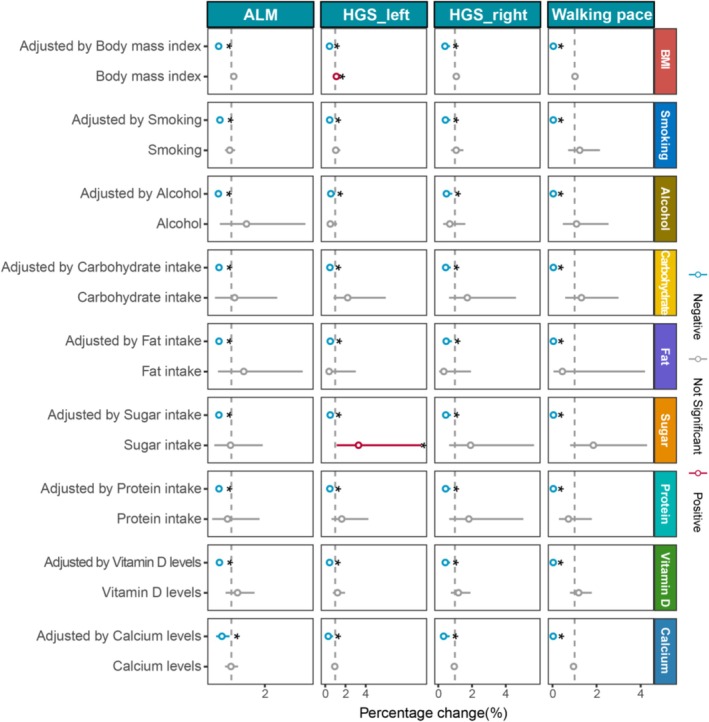
The direct causal effect of physical performance or parameters of sarcopenia on BE by adjusting BMI, smoking traits, alcohol intake, protein intake, lipid intake, carbohydrate intake, sugar intake, as well as vitamin D and calcium. The reported values were calculated by the random effects IVW method. *x*‐axis range is adapted to the distribution of each variable's odds ratios. ALM, appendicular lean mass; BE, Barrett's esophagus; HGS left, left‐hand grip strength; HGS right, right‐hand grip strength; IVW, inverse variance weighted method.

In the multivariable MR analyses, the causal relationship between appendicular lean mass and BE persisted after adjusting for potential confounders. When controlling for BMI, the OR was 0.618 (*p* < 0.001); for smoking traits, the OR was 0.755 (*p* < 0.001); for alcohol intake, the OR was 0.610 (*p* < 0.001); and for protein intake, the OR was 0.628 (*p* < 0.001). Similar inverse associations were observed after controlling for carbohydrate intake (OR = 0.628, *p* < 0.001), fat intake (OR = 0.628, *p* < 0.001), sugar intake (OR = 0.625, *p* < 0.001), vitamin D (OR = 0.738, *p* < 0.001), and calcium intake (OR = 0.640, *p* < 0.001) (Figure 3 and Table S6).

Similarly, the association for left‐hand grip strength remained robust with ORs of 0.432 (*p* < 0.001) after BMI adjustment, 0.792 (*p* = 0.006) after adjusting for smoking traits, 0.558 (*p* = 0.045) after accounting for alcohol intake, and 0.465 (*p* = 0.005) after accounting for protein intake. This association remained significant after controlling for carbohydrate intake (OR = 0.473, *p* = 0.006), fat intake (OR = 0.506, *p* = 0.019), sugar intake (OR = 0.501, *p* = 0.009), vitamin D (OR = 0.753, *p* = 0.003), and calcium intake (OR = 0.307, *p* = 0.013) (Figure 3 and Table S6). Consistent findings were observed for right‐hand grip strength, with adjusted ORs of 0.402 (*p* = 0.001) for BMI, 0.723 (*p* = 0.001) for smoking traits, and 0.487 (*p* = 0.009) for alcohol intake, and 0.439 (*p* = 0.002) for protein intake. Further robustness was confirmed after adjusting for carbohydrate (OR = 0.445, *p* = 0.002), fat (OR = 0.474, *p* = 0.006), sugar intake (OR = 0.450, *p* = 0.002), vitamin D (OR = 0.702, *p* = 0.001), and calcium intake (OR = 0.321, *p* = 0.003) (Figure 3 and Table S6). These findings collectively indicate that both left‐ and right‐hand grip strength exhibit stable and independent inverse associations with BE risk after extensive control for lifestyle and dietary confounders.

Furthermore, walking pace remained inversely associated with the risk of BE after adjusting for BMI (OR = 0.038; *p* < 0.001), smoking (OR = 0.099; *p* < 0.001), alcohol consumption (OR = 0.036; *p* ≤ 0.001), carbohydrate intake (OR = 0.040; *p* < 0.001), fat intake (OR = 0.042; *p* < 0.001), sugar intake (OR = 0.044; *p* < 0.001), protein intake (OR = 0.038; *p* < 0.001), as well as micronutrients including vitamin D (OR = 0.086; *p* < 0.001) and calcium (OR = 0.032; *p* < 0.001). These findings suggest a consistent and potentially independent protective role of higher walking pace against the development of BE (Figure 3 and Table S6).

**FIGURE 4:**
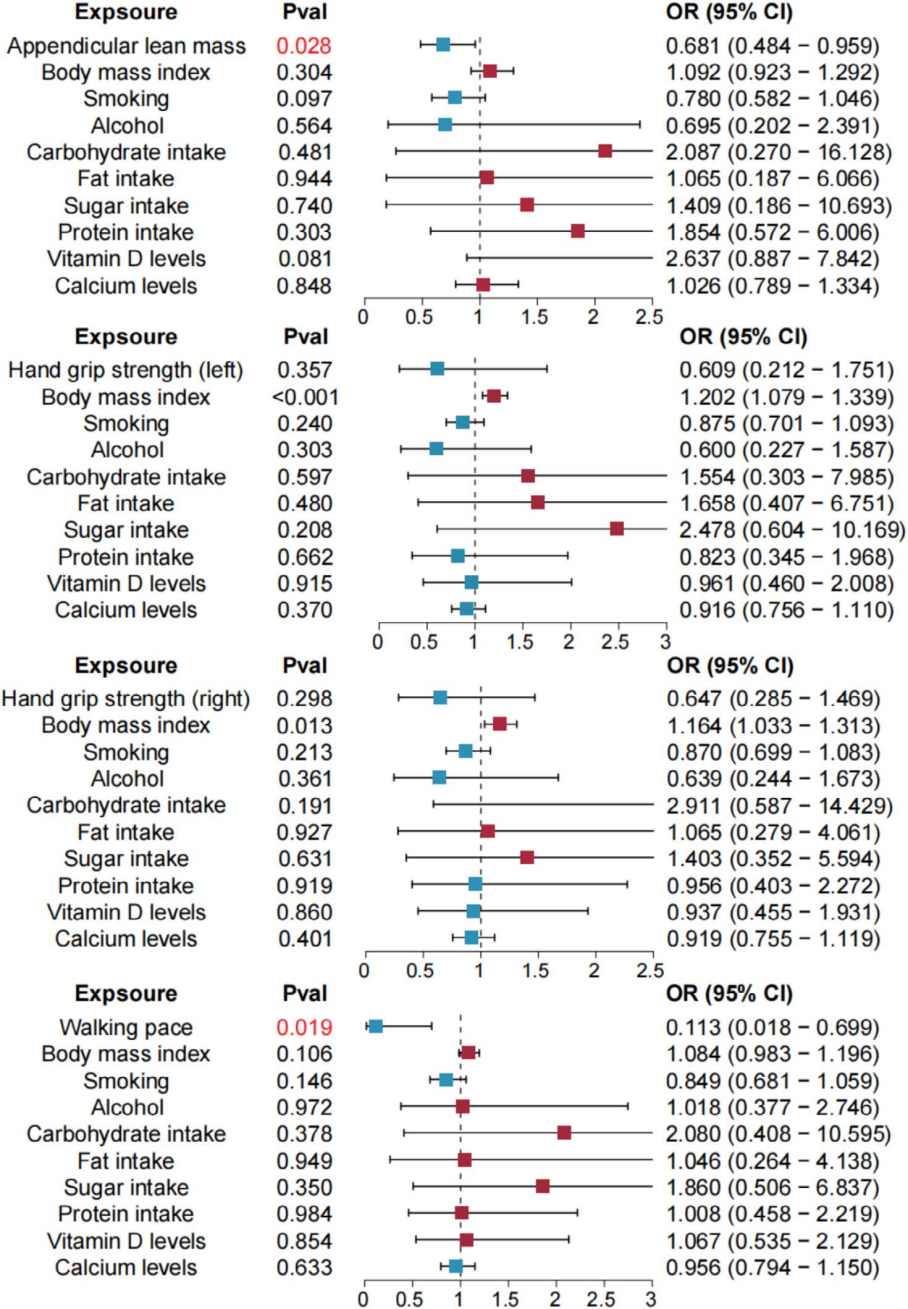
Multivariable Mendelian randomization (MVMR) analysis of sarcopenia‐related traits on BE with all confounders simultaneously included in the model: BMI, smoking, alcohol intake, protein intake, lipid intake, carbohydrate intake, sugar intake, vitamin D, and calcium. Odds ratios and 95% confidence intervals were estimated using the random‐effects IVW method. BE, Barrett's esophagus; IVW, inverse variance weighted method; MVMR, multivariable Mendelian randomization.

In addition, we further performed a comprehensive MVMR analysis by simultaneously including all potential confounders (BMI, smoking, alcohol intake, macronutrient composition, vitamin D, and calcium) in the same model. As shown in Figure 4, the inverse association between appendicular lean mass and BE remained significant (OR = 0.681, 95% CI = 0.484–0.959, *p* = 0.028). Similarly, a higher walking pace was robustly associated with a reduced risk of BE (OR = 0.113, 95% CI = 0.018–0.699, *p* = 0.019). By contrast, the associations for left‐hand grip strength (OR = 0.609, 95% CI = 0.212–1.751, *p* = 0.357) and right‐hand grip strength (OR = 0.647, 95% CI = 0.285–1.469, *p* = 0.298) were attenuated and no longer statistically significant after simultaneous adjustment. These findings suggest that while the protective roles of muscle mass and walking pace are independent and robust, the effects of grip strength may be partially confounded by other lifestyle and dietary factors (Figure 4).

### 3.5 Sensitive Analyses

**FIGURE 5:**
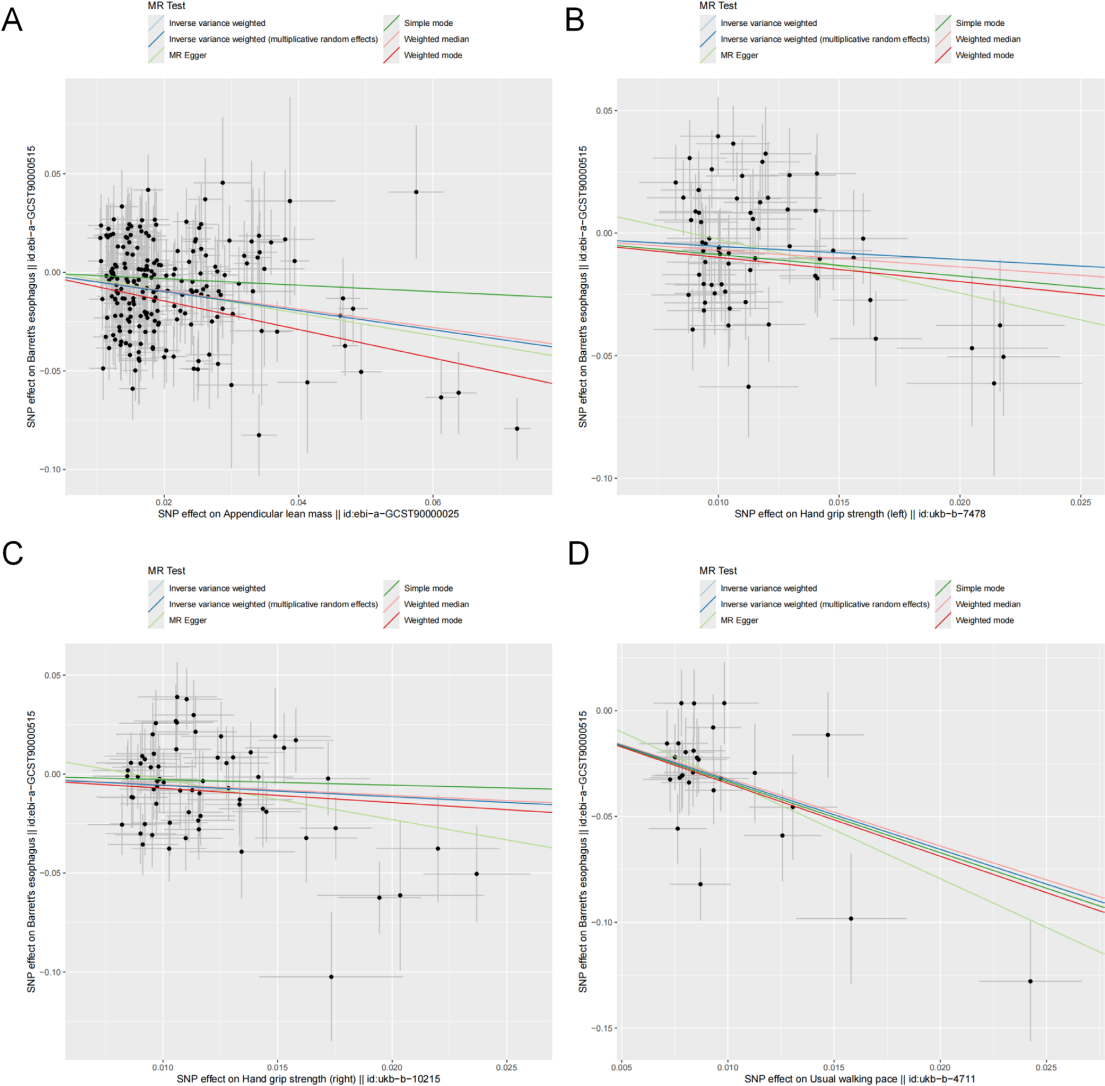
Scatter plot of the association of (A) appendicular lean mass, (B) hand grip strength (left), (C) hand grip strength (right), (D) walking pace and BE. BE, Barrett's esophagus.

**FIGURE 6:**
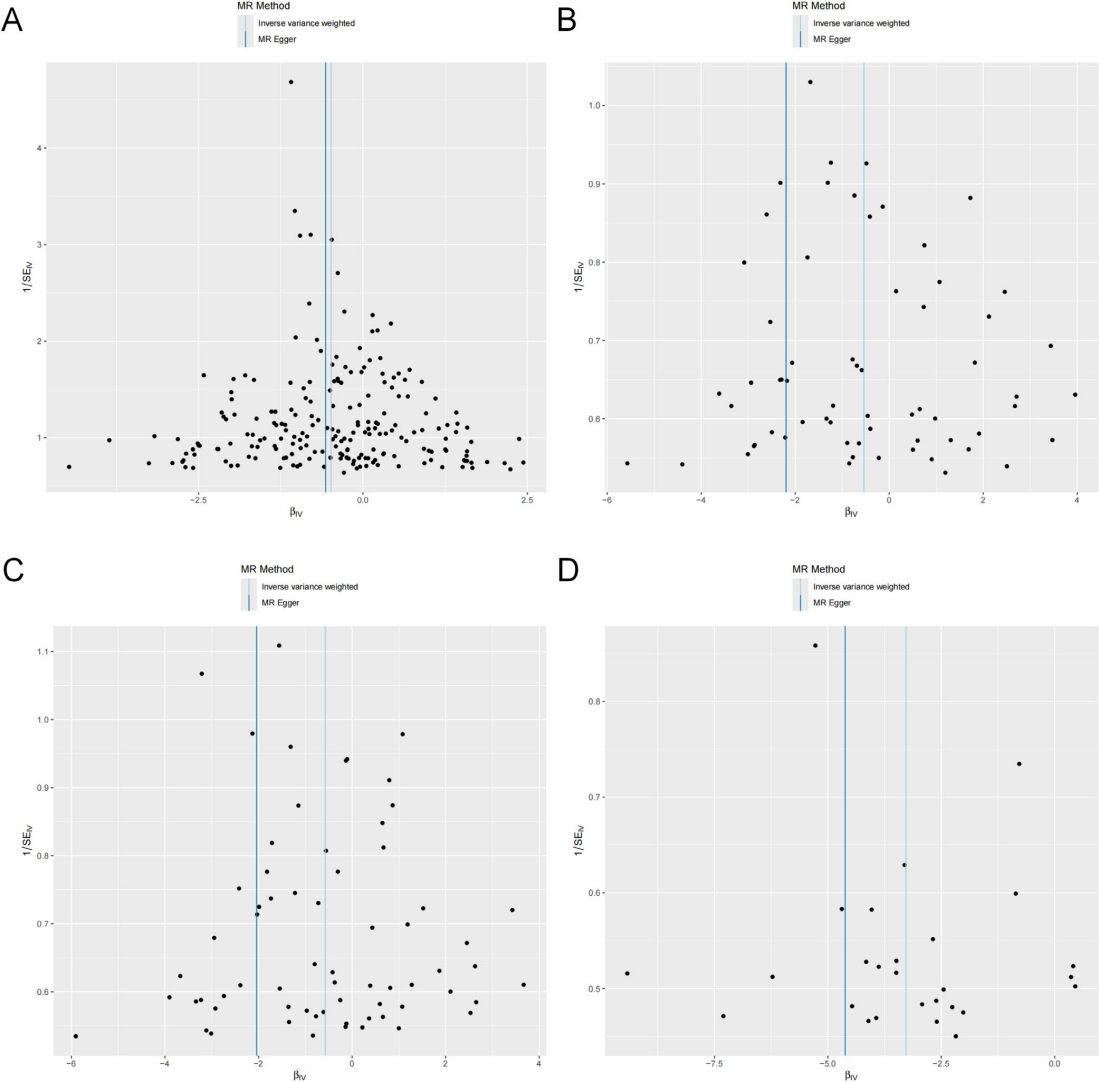
Funnel plot of the association of (A) Appendicular lean mass, (B) Hand grip strength (left), (C) Hand grip strength (right), (D) Walking pace and BE. BE, Barrett's esophagus.

In the univariable MR analyses, MR‐Egger intercept tests did not reveal significant directional pleiotropy for any of the exposures: appendicular lean mass (*p* = 0.573), hand grip strength (left) (*p* = 0.126), hand grip strength (right) (*p* = 0.105), and walking pace (*p* = 0.395) (Table 2). The scatter plot in Figure 5 corroborates these results, and the funnel plot in Figure 6 further confirms the absence of horizontal pleiotropy (Figures S2 and S3). These results support the validity of the instruments and the robustness of the causal inferences. Regarding heterogeneity, heterogeneity tests indicated moderate heterogeneity for most exposures, with *Q*‐test *p*‐values < 0.05 for appendicular lean mass (*p* < 0.001), hand grip strength (left) (*p* < 0.001), and hand grip strength (right) (*p* = 0.001). Walking pace exhibited borderline heterogeneity (*p* = 0.058) (Table 2). These findings suggest potential variability across SNP‐specific causal estimates. Nevertheless, given that the IVW method was implemented under a random‐effects model, the influence of this heterogeneity on the overall estimates is likely mitigated, thus supporting the reliability of the causal inferences.

**TABLE 2:** The result of the heterogeneity and pleiotropy test of sarcopenia and the risk of BE in univariable MR analyses.

Exposure	Heterogeneity		Pleiotropy	
	*Q*	*p*	Egger intercept	*p*
Appendicular lean mass	333.6568	4.33E‐08	0.0020	0.5732
Hand grip strength (left)	109.9000	0.0002	0.0194	0.1261
Hand grip strength (right)	104.6565	0.0013	0.0178	0.1054
Walking pace	38.1733	0.0584	0.0130	0.3945

Abbreviations: BE, Barrett's esophagus; MR, Mendelian randomization.

In the reverse MR analysis, no significant pleiotropy was found (all *p* > 0.05). Moderate heterogeneity was observed for hand grip strength (left), hand grip strength (right), and walking pace (*p* < 0.05), which was addressed with a random‐effects model, supporting the robustness of the causal inferences (Table S7).

In sensitivity analyses of the multivariable MR models, no evidence of horizontal pleiotropy was detected by MR‐Egger regression across all adjustments, including BMI, smoking, alcohol intake, protein intake, lipid intake, carbohydrate intake, sugar intake, as well as vitamin D and calcium (all *p*_pleiotropy > 0.05; Table 3). While some degree of heterogeneity (*p*_heterogeneity < 0.05) was observed under several models—particularly for appendicular lean mass and hand grip strength—these results remained consistent with the primary analysis. The use of random‐effects models in our multivariable MR framework further mitigates the influence of such heterogeneity, supporting the overall robustness and credibility of our findings. In addition, we evaluated instrument strength across all exposures by calculating F‐statistics in the multivariable MR framework (Table S8). While several instruments exhibited F‐statistics below the conventional threshold of 10, indicating potential weak instrument bias, the use of random‐effects IVW models in multivariable MR allows for heterogeneity in instrument strength and provides more conservative and robust estimates. Furthermore, in the comprehensive MVMR models with all confounders simultaneously included, no evidence of horizontal pleiotropy was detected (all *p* for Egger intercept > 0.05), while significant heterogeneity was observed for several traits. Similar to the single‐confounder MVMR analyses, the application of random‐effects models in the comprehensive MVMR framework accounted for this variability, yielding estimates that were stable and in line with the main results (Table 4).

**TABLE 3:** The result of heterogeneity and pleiotropy test of sarcopenia and risk of BE in multivariable MR analyses.

Confounders	Method	Appendicular lean mass		Hand grip strength (left)		Hand grip strength (right)		Walking pace	
Adjusted by BMI	Heterogeneity	Cochrane's *Q* = 361.641	*p*_het_ < 0.001	Cochrane's *Q* = 200.141	*p*_het_ < 0.001	Cochrane's *Q* = 158.955	*p*_het_ < 0.001	Cochrane's *Q* = 37.512	*p*_het_ = 0.108
	Pleiotropy‐Egger intercept	Intercept = 6.37E‐03	*p*_ple_ = 0.098	Intercept = −2.97E‐03	*p*_ple_ = 0.344	Intercept = −6.26E‐03	*p*_ple_ = 0.173	Intercept = 5.84E‐03	*p*_ple_ = 0.349
Adjusted by smoking	Heterogeneity	Cochrane's *Q* = 363.746	*p*_het_ < 0.001	Cochrane's *Q* = 162.552	*p*_het_ < 0.001	Cochrane's *Q* = 167.694	*p*_het_ < 0.001	Cochrane's *Q* = 41.098	*p*_het_ = 0.012
	Pleiotropy‐Egger intercept	Intercept = 8.02E‐04	*p*_ple_ = 0.746	Intercept = 1.12E‐03	*p*_ple_ = 0.843	Intercept = 3.04E‐04	*p*_ple_ = 0.955	Intercept = 9.16E‐03	*p*_ple_ = 0.240
Adjusted by alcohol	Heterogeneity	Cochrane's *Q* = 386.448	*p*_het_ < 0.001	Cochrane's *Q* = 158.300	*p*_het_ < 0.001	Cochrane's *Q* = 170.596	*p*_het_ < 0.001	Cochrane's *Q* = 50.353	*p*_het_ = 0.008
	Pleiotropy‐Egger intercept	Intercept = 5.74E‐03	*p*_ple_ = 0.137	Intercept = −2.22E‐03	*p*_ple_ = 0.601	Intercept = −3.83E‐03	*p*_ple_ = 0.357	Intercept = 5.48E‐03	*p*_ple_ = 0.320
Adjusted by carbohydrate intake	Heterogeneity	Cochrane's *Q* = 407.005	*p*_het_ < 0.001	Cochrane's *Q* = 157.907	*p*_het_ < 0.001	Cochrane's *Q* = 172.057	*p*_het_ < 0.001	Cochrane's *Q* = 40.664	*p*_het_ = 0.074
	Pleiotropy‐Egger intercept	Intercept = 7.00E‐04	*p*_ple_ = 0.766	Intercept = 1.50E‐02	*p*_ple_ = 0.118	Intercept = −1.08E‐03	*p*_ple_ = 0.786	Intercept = −8.56E‐04	*p*_ple_ = 0.867
Adjusted by fat intake	Heterogeneity	Cochrane's *Q* = 404.070	*p*_het_ < 0.001	Cochrane's *Q* = 157.569	*p*_het_ < 0.001	Cochrane's *Q* = 159.099	*p*_het_ < 0.001	Cochrane's *Q* = 34.252	*p*_het_ = 0.080
	Pleiotropy‐Egger intercept	Intercept = −7.70E‐04	*p*_ple_ = 0.741	Intercept = −3.98E‐03	*p*_ple_ = 0.380	Intercept = −2.94E‐03	*p*_ple_ = 0.542	Intercept = 2.24E‐03	*p*_ple_ = 0.742
Adjusted by sugar intake	Heterogeneity	Cochrane's *Q* = 403.916	*p*_het_ < 0.001	Cochrane's *Q* = 146.003	*p*_het_ < 0.001	Cochrane's *Q* = 164.017	*p*_het_ < 0.001	Cochrane's *Q* = 34.864	*p*_het_ = 0.142
	Pleiotropy‐Egger intercept	Intercept = 1.96E‐03	*p*_ple_ = 0.368	Intercept = 5.39E‐03	*p*_ple_ = 0.151	Intercept = −2.57E‐03	*p*_ple_ = 0.529	Intercept = 4.41E‐03	*p*_ple_ = 0.381
Adjusted by protein intake	Heterogeneity	Cochrane's *Q* = 406.654	*p*_het_ < 0.001	Cochrane's *Q* = 165.475	*p*_het_ < 0.001	Cochrane's *Q* = 168.870	*p*_het_ < 0.001	Cochrane's *Q* = 46.685	*p*_het_ = 0.015
	Pleiotropy‐Egger intercept	Intercept = 2.73E‐03	*p*_ple_ = 0.241	Intercept = 2.46E‐03	*p*_ple_ = 0.573	Intercept = −2.63E‐04	*p*_ple_ = 0.949	Intercept = 7.45E‐03	*p*_ple_ = 0.170
Adjusted by vitamin D levels	Heterogeneity	Cochrane's *Q* = 363.023	*p*_het_ < 0.001	Cochrane's *Q* = 168.394	*p*_het_ < 0.001	Cochrane's *Q* = 179.170	*p*_het_ < 0.001	Cochrane's *Q* = 52.750	*p*_het_ = 0.008
	Pleiotropy‐Egger intercept	Intercept = 2.49E‐03	*p*_ple_ = 0.128	Intercept = 2.15E‐03	*p*_ple_ = 0.531	Intercept = −1.88E‐03	*p*_ple_ = 0.571	Intercept = 3.39E‐03	*p*_ple_ = 0.428
Adjusted by calcium levels	Heterogeneity	Cochrane's *Q* = 198.751	*p*_het_ < 0.001	Cochrane's *Q* = 214.417	*p*_het_ < 0.001	Cochrane's *Q* = 232.217	*p*_het_ < 0.001	Cochrane's *Q* = 165.299	*p*_het_ = 0.001
	Pleiotropy‐Egger intercept	Intercept = 3.46E‐03	*p*_ple_ = 0.401	Intercept = 9.44E‐04	*p*_ple_ = 0.807	Intercept = 3.12E‐03	*p*_ple_ = 0.390	Intercept = −2.24E‐03	*p*_ple_ = 0.525

Abbreviations: BE, Barrett's esophagus; BMI, body mass index; MR, Mendelian randomization.

**TABLE 4:** The result of the heterogeneity and pleiotropy test of sarcopenia and the risk of BE in multivariable MR analyses with all confounders simultaneously included in the MVMR model.

Exposure	Heterogeneity		Pleiotropy	
	*Q*	*p*	Egger intercept	*p*
Appendicular lean mass with all confounders simultaneously included in the MVMR model	127.718	< 0.001	−0.002	0.615
Hand grip strength (left) with all confounders simultaneously included in the MVMR model	136.767	0.003	−0.002	0.627
Hand grip strength (right) with all confounders simultaneously included in the MVMR model	162.115	< 0.001	−0.001	0.677
Walking pace with all confounders simultaneously included in the MVMR model	141.567	0.003	−0.003	0.313

Abbreviations: BE, Barrett's esophagus; MR, Mendelian randomization; MVMR, multivariable MR.

## 4 Discussion

Sarcopenia has emerged as a critical factor in understanding various health conditions, (Ata et al. 2020; Beaudart et al. 2017) including its potential link to BE. (Kim et al. 2019; Imagama et al. 2019) Several studies have explored the relationship between sarcopenia and the incidence of BE. Early research may have suggested a connection between sarcopenia and an increased susceptibility to BE. (Kim et al. 2019; Imagama et al. 2019) However, unlike traditional observational studies, methods like MR analysis offer a more refined approach, less prone to confounding variables or reverse causality. Therefore, employing such analyses could provide valuable insights into the complex causal relationship between sarcopenia and the risk of developing BE.

This analysis represents the first MR study exploring the causal relationship between sarcopenia and BE. In our multivariable MR analysis, we selected BMI (Seidel et al. 2009), smoking traits, alcohol consumption (Kim et al. 2019), protein intake, lipid intake, carbohydrate intake, sugar intake, as well as vitamin D and calcium levels (Calvani et al. 2023) as covariates based on their known biological and epidemiological relevance to both sarcopenia and Barrett's esophagus. Specifically, these factors are recognized for their pleiotropic influences on metabolic homeostasis, skeletal muscle maintenance, and esophageal pathology. By adjusting for these variables, we aimed to better isolate the direct causal effects of sarcopenia‐related traits on BE risk while minimizing confounding bias. Our findings suggest that physical performance and sarcopenia parameters (including appendicular lean mass, hand grip strength, and walking pace) are inversely associated with BE risk, indicating a protective effect of greater muscle mass and strength. After adjusting for BMI, smoking, alcohol consumption, protein intake, lipid intake, carbohydrate intake, sugar intake, as well as vitamin D and calcium levels, appendicular lean mass continued to show a negative association with BE incidence. The multivariable MR analysis for hand grip strength revealed that the causal relationship between both left and right hand grip strength and BE remained significant, even after controlling for potential confounders. Similarly, the causal effect of walking pace on BE maintained after adjusting for these four confounders. Consistent with our MR findings, Shiro et al. reported an increased risk of BE in sarcopenia patients (Imagama et al. 2019). Unlike previous observational studies, however, the results from our MR analysis may offer more robust causal insights due to the absence of confounding factors and reverse causality.

In the comprehensive MVMR models with all confounders simultaneously included, both appendicular lean mass (OR = 0.681, *p* = 0.028) and walking pace (OR = 0.113, *p* = 0.019) remained significantly protective, highlighting the robustness of these associations. In contrast, the associations for hand grip strength were attenuated and lost statistical significance, suggesting that the observed effects of grip strength may be partially confounded by lifestyle or nutritional factors. From a sarcopenia‐defining perspective, appendicular lean mass and walking pace may serve as more representative indicators than grip strength. Appendicular lean mass directly quantifies skeletal muscle mass in the extremities, which is the structural foundation of sarcopenia and a key diagnostic criterion in international consensus definitions (Kim et al. 2019; Imagama et al. 2019). Reduced appendicular lean mass reflects the progressive loss of muscle tissue that underpins metabolic dysfunction, chronic inflammation, and frailty, all of which are biologically relevant to Barrett's esophagus. Walking pace, on the other hand, captures the integrated output of multiple systems, including lower‐limb muscle strength, balance, cardiopulmonary fitness, and neural control. As such, it reflects the global functional consequences of sarcopenia rather than muscle quantity alone, and has been consistently linked to adverse outcomes such as disability, hospitalization, and mortality. By contrast, hand grip strength, although widely used as a simple screening tool, primarily reflects upper‐limb muscle performance. Its value can be confounded by body composition (e.g., higher BMI or fat mass artificially augmenting grip force), musculoskeletal comorbidities (such as osteoarthritis or tendon disorders), and lifestyle or occupational factors that disproportionately train upper‐limb muscles. Moreover, grip strength does not fully capture lower‐limb or trunk muscle function, which are more directly relevant to esophageal physiology, abdominal pressure regulation, and systemic metabolic health. These limitations may explain why the associations of grip strength with BE risk were attenuated in our fully adjusted MVMR models, whereas appendicular lean mass and walking pace retained robust protective effects. Together, these considerations suggest that muscle mass and overall physical performance may represent more direct and stable protective factors for BE risk than grip strength alone.

There are several potential mechanisms that could explain the causal link between sarcopenia and the development of BE: (a) Impact on metabolism and gastrointestinal motility: The decrease in muscle mass characteristic of sarcopenia can lead to changes in overall metabolism and energy expenditure, (Argilés et al. 2016) which may affect gastrointestinal motility. (Martin‐Gallausiaux et al. 2021; Ryan et al. 2012) These alterations in motility could impair the normal movement of food through the digestive tract, leading to delayed gastric emptying or inefficient digestion. This delay in transit time could increase the risk of BE by prolonging the exposure of the esophagus to stomach acid and digestive enzymes. (b) Visceral fat accumulation and relaxation of the LES (lower esophageal sphincter): Sarcopenia is often associated with shifts in body composition, such as an increase in visceral fat. (Choi 2016; Li et al. 2022; Wannamethee and Atkins 2015) Higher levels of visceral fat have been linked to inflammation and metabolic dysfunction, (Hall et al. 2021; Lyon et al. 2003; Zahid et al. 2016) which may contribute to the relaxation of the LES, thereby exacerbating BE symptoms. (Kandulski and Malfertheiner 2011) (c) BE is primarily driven by chronic gastroesophageal reflux, which in turn is influenced by several mechanical and functional factors. Sarcopenia may contribute to BE development by impairing diaphragmatic contractility, reducing lower esophageal sphincter competence, and diminishing esophageal peristalsis—all of which are essential for reflux clearance. In particular, loss of core and postural muscle strength could alter intra‐abdominal pressure dynamics and promote more frequent or severe reflux episodes, a known precursor to BE. However, the exact mechanisms underlying the relationship between sarcopenia and BE remain to be fully elucidated. Further research is needed to explore the complex interplay between sarcopenia and BE and such studies may lead to novel therapeutic strategies for both preventing and managing BE. From a clinical standpoint, our findings suggest that patients with sarcopenia—especially elderly individuals with longstanding reflux symptoms—may warrant closer evaluation for BE, even in the absence of obesity or other classic risk factors. Incorporating simple physical performance assessments, such as grip strength testing or gait speed measurements, into GERD management pathways may help identify high‐risk individuals for earlier endoscopic screening or preventive counseling.

This study presents several strengths and limitations. A major strength is the implementation of MR analysis, which minimizes residual confounding and reverse causality, thereby offering a more robust evaluation of the link between sarcopenia and BE. Nonetheless, some limitations should be considered. First, while it is challenging to completely rule out directional pleiotropy in any MR study, our MR‐Egger intercept tests did not suggest the presence of such effects, and the sensitivity analyses yielded consistent results. Second, since most of the GWAS data used were from individuals of European descent, the generalizability of our findings to other populations is limited. Future MR analyses based on more diverse ancestry datasets are needed to validate these results across different ethnic groups. Third, although the comprehensive MVMR model included nine potential confounders, this approach enabled us to assess the independence of the associations. The inverse associations for appendicular lean mass and walking pace remained significant, whereas those for hand grip strength were attenuated, possibly due to shared pathways with metabolic or lifestyle factors. These findings collectively suggest that muscle‐related traits exert protective effects against BE. In addition, caution is warranted when interpreting the causal estimates in the multivariable MR analyses, as some instrumental variables exhibited *F*‐statistics below the conventional threshold of 10 (Table S8), suggesting a potential for weak instrument bias. However, the application of random‐effects IVW models in these analyses helps mitigate this issue by allowing for heterogeneity among instruments, thus yielding more conservative and reliable estimates even in the presence of variable instrument strength.

## 5 Conclusions

This multivariable MR analysis demonstrated a causal link between sarcopenia‐related physical performance metrics—including appendicular lean mass, hand grip strength, and walking pace—and BE. These insights offer healthcare providers a basis for designing more targeted interventions, screenings, and treatment strategies for individuals with sarcopenia, acknowledging a potentially reduced risk for BE in individuals with preserved muscle mass and function, whereas sarcopenia itself may increase susceptibility. Nonetheless, establishing a definitive causal relationship is complex, and these results warrant further confirmation through extensive, long‐term clinical studies.

## Author Contributions

J.Z., D.E., Y.T.: Conceptualization, writing – original draft, software, resources, methodology, data curation. Y.L., P.F.: Writing – review and editing, software, resources, methodology, data curation. Z.Y., Y.Y.: Writing – review and editing, visualization, funding acquisition.

## Ethics Statement

All analyses were based on previously published studies; thus no ethical approval and patient consent are required.

## Consent

The authors have nothing to report.

## Conflicts of Interest

The authors declare no conflicts of interest.

## Supporting information


**Figure S1:** Associations of genetically predicted BE with physical performance or parameters of sarcopenia. The reported values were calculated by the fixed effects IVW method. OR, odds ratio; CI, confidence interval; IVW, inverse variance weighted method; BE, Barrett's esophagus.
**Figure S2:** Forest plot showing the association of (A) Appendicular lean mass, (B) Hand grip strength (left), (C) Hand grip strength (right), and (D) Walking pace with BE. BE, Barrett's esophagus.
**Figure S3:** Leave‐one‐out sensitivity analysis of the association of (A) Appendicular lean mass, (B) Hand grip strength (left), (C) Hand grip strength (right), and (D) Walking pace with BE. BE, Barrett's esophagus.

**Table S1:** Single nucleotide polymorphisms used as instrumental variables in the Mendelian randomization analyses of Appendicular lean mass.
**Table S2:** Single nucleotide polymorphisms used as instrumental variables in the Mendelian randomization analyses of Hand grip strength (left).
**Table S3:** Single nucleotide polymorphisms used as instrumental variables in the Mendelian randomization analyses of Hand grip strength (right).
**Table S4:** Single nucleotide polymorphisms used as instrumental variables in the Mendelian randomization analyses of Walking pace.
**Table S5:** Instrumental variables of Barrett's esophagus in reverse MR analyses.
**Table S6:** The result of the direct causal effect of physical performance or parameters of sarcopenia on BE by adjusting BMI, smoking traits, alcohol intake, protein intake, lipid intake, carbohydrate intake, sugar intake, as well as vitamin D and calcium. The reported values were calculated by the random effects IVW method. OR, odds ratio; CI, confidence interval; IVW, inverse variance weighted method; BE, Barrett's esophagus.
**Table S7:** The result of heterogeneity and pleiotropy test of BE and risk of sarcopenia in reverse MR analyses.
**Table S8:** The result of the *F* statistic of sarcopenia and risk of BE in multivariable MR analyses.

## Data Availability

All data used in this study was obtained from publicly available GWAS summary datasets.
