# Supplementary material for: Genetically Predicted Sarcopenia Traits and the Risk of Barrett's Esophagus
Source: Food Sci Nutr. 2025 Nov 13;13(11):e71148. doi: 10.1002/fsn3.71148 (PMC12614087; doi:10.1002/fsn3.71148)
Supplement: Supplementary file 1 — Figure S1: Associations of genetically predicted BE with physical performance or parameters of sarcopenia. The reported values were calculated by the fixed effects IVW method. OR, odds ratio; CI, confidence interval; IVW, inverse variance weighted method; BE, Barrett's esophagus. Figure S2: Forest plot showing the association of (A) Appendicular lean mass, (B) Hand grip strength (left), (C) Hand grip strength (right), and (D) Walking pace with BE. BE, Barrett's esophagus. Figure S3: Leave‐one‐out sensitivity analysis of the association of (A) Appendicular lean mass, (B) Hand grip strength (left), (C) Hand grip strength (right), and (D) Walking pace with BE. BE, Barrett's esophagus. [file FSN3-13-e71148-s001.docx]

# Supplementary materials:

# Supplemental Figure S1 Associations of genetically predicted BE with physical performance or parameters of sarcopenia. The reported values were calculated by the fixed effects IVW method. OR, odds ratio; CI, confidence interval; IVW, inverse variance weighted method; BE, Barrett's esophagus.

# Supplemental Figure S2. Forest plot showing the association of (A) Appendicular lean mass, (B) Hand grip strength (left), (C) Hand grip strength (right), and (D) Walking pace with BE. BE, Barrett's esophagus.

**Supplemental Figure S3.** Leave-one-out sensitivity analysis of the association of (A) Appendicular lean mass, (B) Hand grip strength (left), (C) Hand grip strength (right), and (D) Walking pace with BE. BE, Barrett's esophagus.


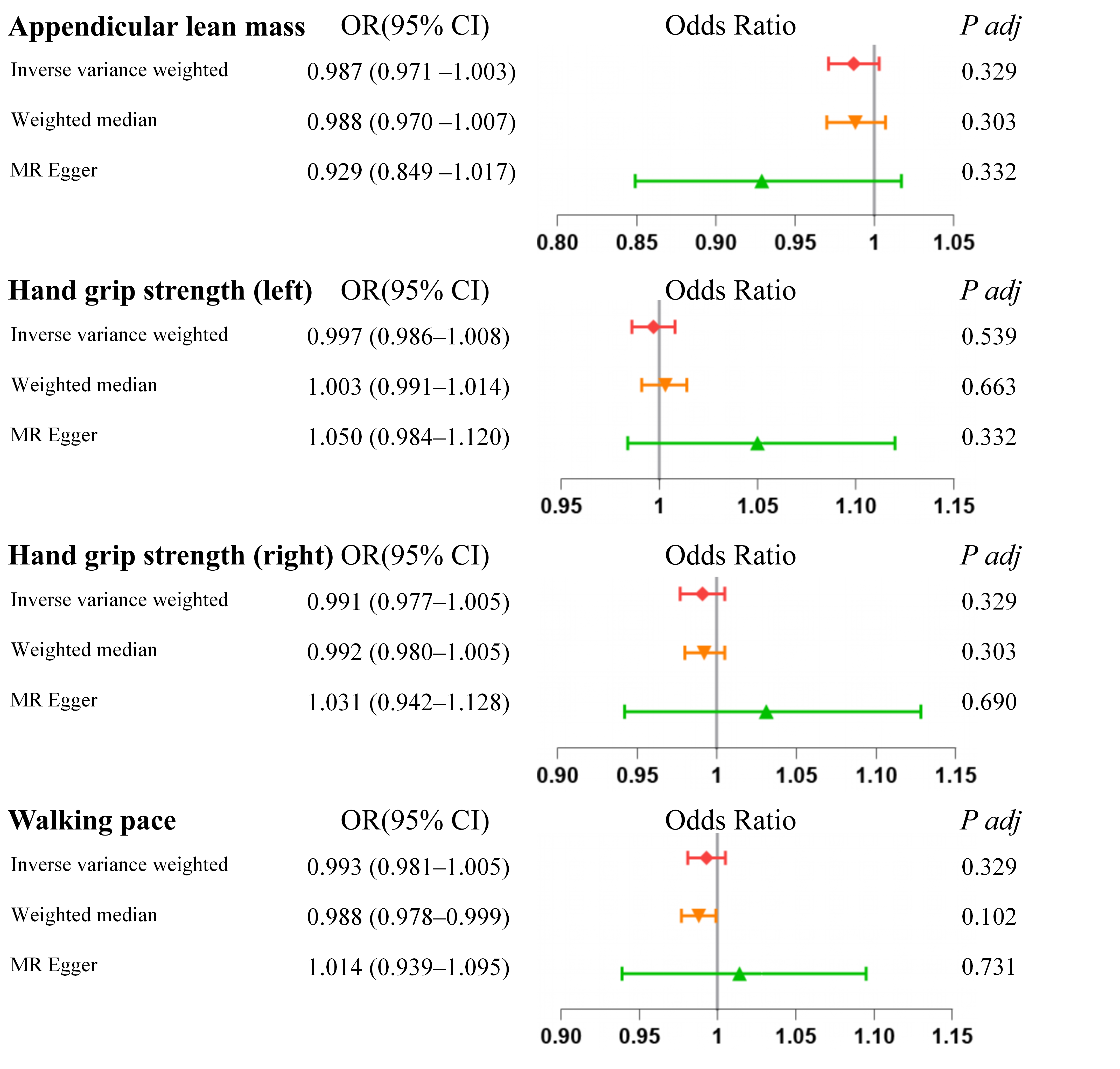


# Supplemental Figure S1 Associations of genetically predicted BE with physical performance or parameters of sarcopenia. The reported values were calculated by the fixed effects IVW method. OR, odds ratio; CI, confidence interval; IVW, inverse variance weighted method; BE, Barrett's esophagus.


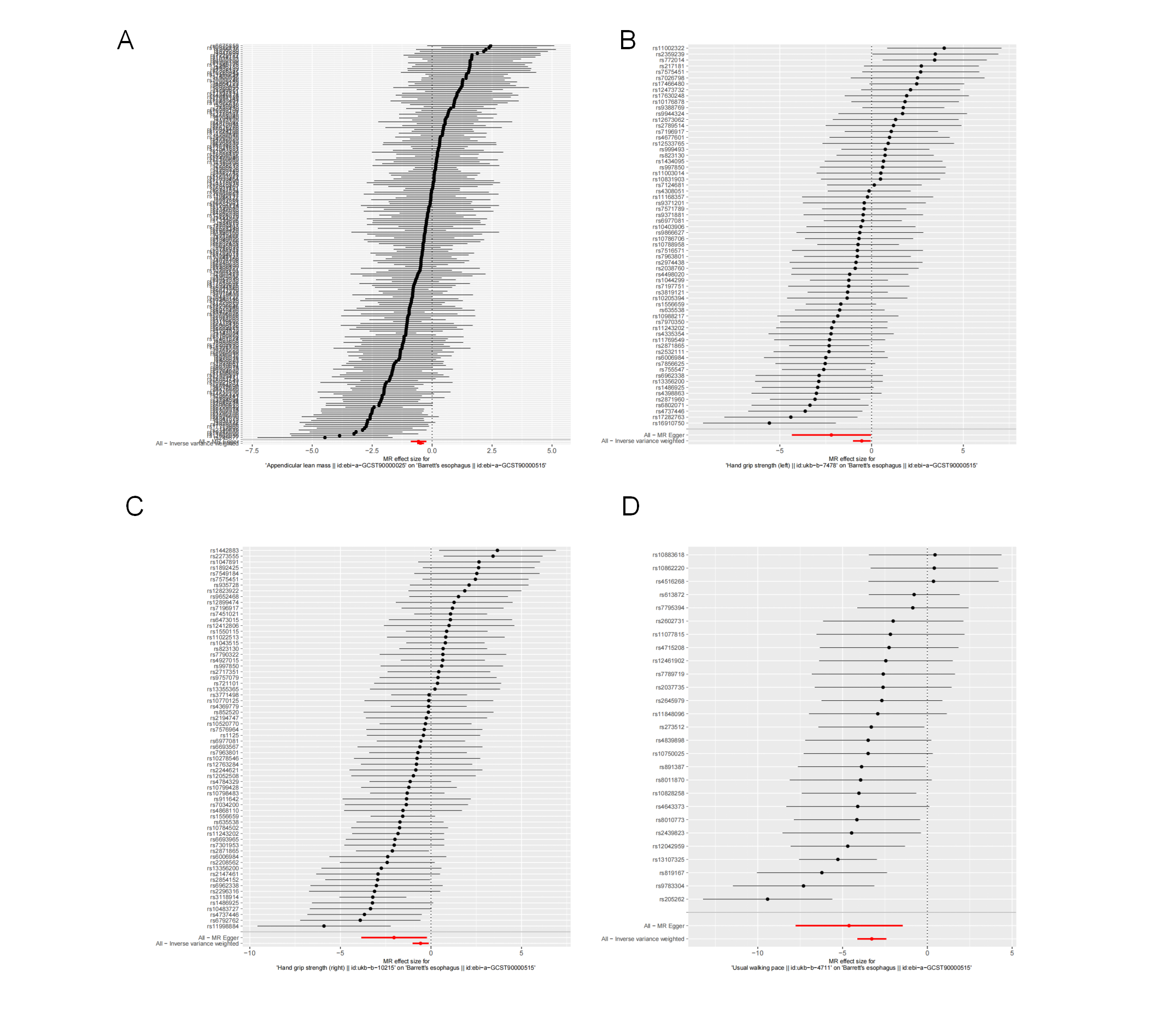


# Supplemental Figure S2. Forest plot showing the association of (A) Appendicular lean mass, (B) Hand grip strength (left), (C) Hand grip strength (right), and (D) Walking pace with BE. BE, Barrett's esophagus.


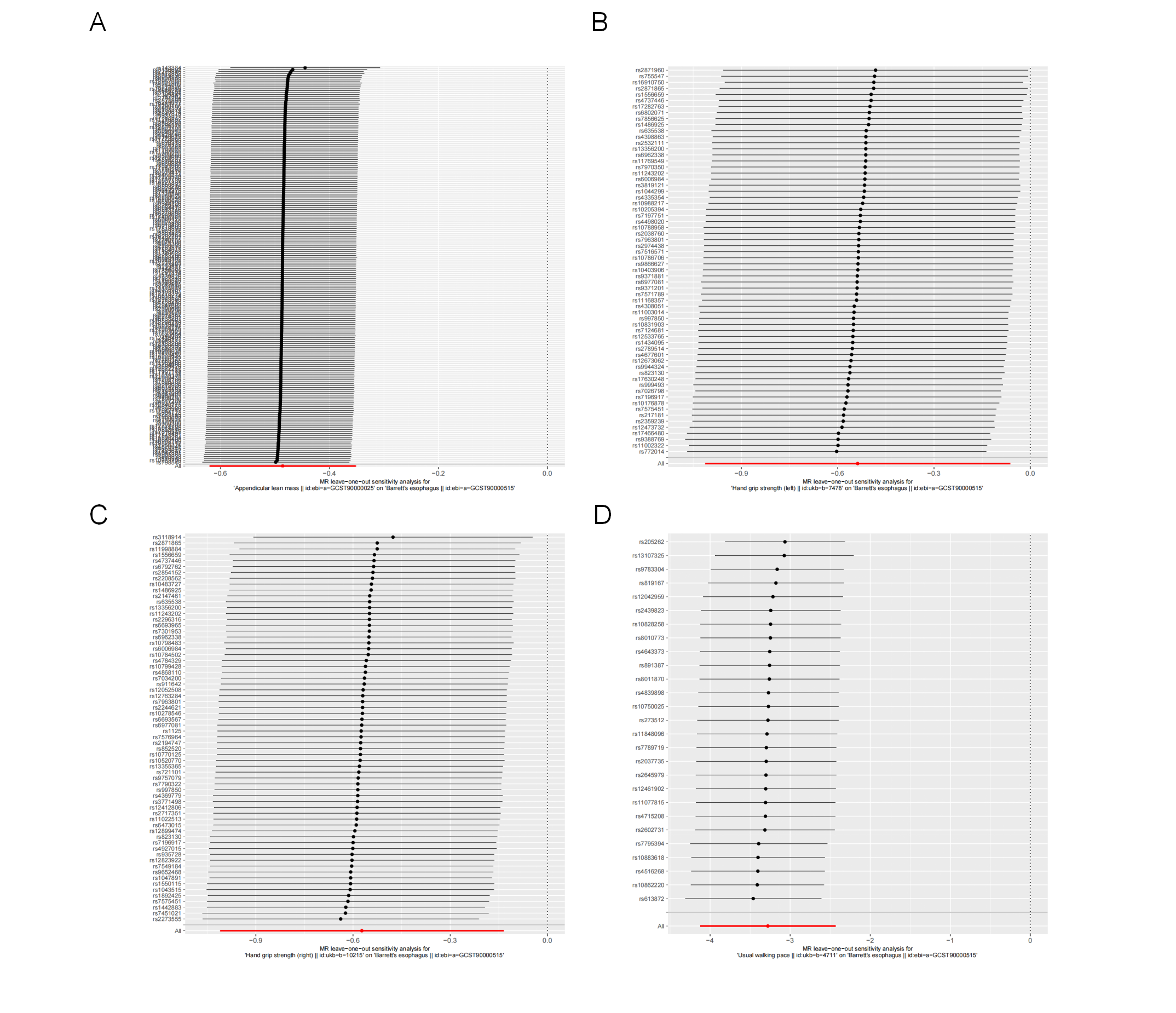
**Supplemental Figure S3.** Leave-one-out sensitivity analysis of the association of (A) Appendicular lean mass, (B) Hand grip strength (left), (C) Hand grip strength (right), and (D) Walking pace with BE. BE, Barrett's esophagus.
