# Supplementary material for: Genetically Predicted Sarcopenia Traits and the Risk of Barrett's Esophagus
Source: Food Sci Nutr. 2025 Nov 13;13(11):e71148. doi: 10.1002/fsn3.71148 (PMC12614087; doi:10.1002/fsn3.71148)
Supplement: Supplementary file 2 — Table S1: Single nucleotide polymorphisms used as instrumental variables in the Mendelian randomization analyses of Appendicular lean mass. Table S2: Single nucleotide polymorphisms used as instrumental variables in the Mendelian randomization analyses of Hand grip strength (left). Table S3: Single nucleotide polymorphisms used as instrumental variables in the Mendelian randomization analyses of Hand grip strength (right). Table S4: Single nucleotide polymorphisms used as instrumental variables in the Mendelian randomization analyses of Walking pace. Table S5: Instrumental variables of Barrett's esophagus in reverse MR analyses. Table S6: The result of the direct causal effect of physical performance or parameters of sarcopenia on BE by adjusting BMI, smoking traits, alcohol intake, protein intake, lipid intake, carbohydrate intake, sugar intake, as well as vitamin D and calcium. The reported values were calculated by the random effects IVW method. OR, odds ratio; CI, confidence interval; IVW, inverse variance weighted method; BE, Barrett's esophagus. Table S7: The result of heterogeneity and pleiotropy test of BE and risk of sarcopenia in reverse MR analyses. Table S8: The result of the F statistic of sarcopenia and risk of BE in multivariable MR analyses. [file FSN3-13-e71148-s002.docx]

# Supplementary materials:

**Supplemental Table S1.** Single nucleotide polymorphisms used as instrumental variables in the Mendelian randomization analyses of Appendicular lean mass.

**Supplemental Table S2.** Single nucleotide polymorphisms used as instrumental variables in the Mendelian randomization analyses of Hand grip strength (left)

**Supplemental Table S3.** Single nucleotide polymorphisms used as instrumental variables in the Mendelian randomization analyses of Hand grip strength (right)

**Supplemental Table S4.** Single nucleotide polymorphisms used as instrumental variables in the Mendelian randomization analyses of Walking pace

**Supplemental Table S5.** Instrumental variables of Barrett's esophagus in reverse MR analyses

**Supplemental Table S6.** The result of the direct causal effect of physical performance or parameters of sarcopenia on BE by adjusting BMI, smoking traits, alcohol intake, protein intake, lipid intake, carbohydrate intake, sugar intake, as well as vitamin D and calcium. The reported values were calculated by the random effects IVW method. *OR, odds ratio; CI, confidence interval; IVW, inverse variance weighted method; BE, Barrett's esophagus.*

**Supplemental Table S7.** The result of heterogeneity and pleiotropy test of BE and risk of sarcopenia in reverse MR analyses

**Supplemental Table S8.** The result of the F statistic of sarcopenia and risk of BE in multivariable MR analyses.

**Supplemental Table S1.** Single nucleotide polymorphisms used as instrumental variables in the Mendelian randomization analyses of Appendicular lean mass.

| **SNP** | **Chr** | **EA** | **NEA** | **Beta** | **SE** | **F** |
| --- | --- | --- | --- | --- | --- | --- |
| rs10005035 | 4 | G | C | -0.018 | 0.002 | 69.444 |
| rs10202701 | 2 | T | C | 0.023 | 0.002 | 142.739 |
| rs10221831 | 2 | T | C | 0.030 | 0.005 | 32.040 |
| rs10225945 | 7 | G | A | -0.015 | 0.003 | 31.532 |
| rs10242866 | 7 | T | C | 0.016 | 0.002 | 68.279 |
| rs10283100 | 8 | G | A | 0.058 | 0.004 | 196.683 |
| rs1035583 | 2 | A | G | 0.015 | 0.002 | 60.676 |
| rs1047891 | 2 | A | C | 0.023 | 0.002 | 135.722 |
| rs10483727 | 14 | C | T | -0.037 | 0.002 | 375.134 |
| rs1063582 | 8 | G | T | -0.019 | 0.002 | 70.712 |
| rs10748128 | 12 | T | G | 0.026 | 0.002 | 162.562 |
| rs10796828 | 11 | G | T | 0.015 | 0.002 | 59.290 |
| rs10815274 | 9 | C | A | 0.012 | 0.002 | 42.593 |
| rs10822117 | 10 | G | A | -0.018 | 0.002 | 64.000 |
| rs10832963 | 11 | G | T | -0.020 | 0.002 | 85.142 |
| rs10845408 | 12 | T | C | 0.026 | 0.002 | 162.562 |
| rs10858246 | 9 | C | G | -0.019 | 0.002 | 88.360 |
| rs10948 | 19 | T | G | -0.025 | 0.002 | 158.759 |
| rs11014285 | 10 | A | G | 0.034 | 0.003 | 173.023 |
| rs11042717 | 11 | C | T | -0.029 | 0.002 | 232.963 |
| rs11175919 | 12 | A | G | 0.035 | 0.006 | 34.990 |
| rs11198591 | 10 | A | G | 0.015 | 0.002 | 54.760 |
| rs11210892 | 1 | A | G | 0.012 | 0.002 | 34.810 |
| rs11243202 | 6 | C | T | 0.030 | 0.002 | 252.642 |
| rs11590254 | 1 | T | A | 0.019 | 0.002 | 86.490 |
| rs11633371 | 15 | T | G | 0.022 | 0.002 | 129.240 |
| rs1168768 | 12 | T | C | 0.033 | 0.006 | 30.618 |
| rs11778491 | 8 | C | G | -0.025 | 0.002 | 126.051 |
| rs1190540 | 14 | G | A | 0.013 | 0.002 | 35.431 |
| rs11959466 | 5 | T | C | 0.038 | 0.004 | 81.859 |
| rs1202186 | 7 | T | C | -0.012 | 0.002 | 36.000 |
| rs12188208 | 5 | C | A | -0.020 | 0.002 | 78.564 |
| rs12230946 | 12 | A | G | 0.027 | 0.003 | 67.439 |
| rs12340775 | 9 | A | G | -0.029 | 0.004 | 44.548 |
| rs12461874 | 19 | A | C | -0.018 | 0.002 | 74.288 |
| rs12483401 | 21 | C | T | -0.039 | 0.007 | 33.363 |
| rs12519407 | 5 | C | A | 0.018 | 0.002 | 67.688 |
| rs12533452 | 7 | T | C | 0.024 | 0.003 | 83.090 |
| rs12541381 | 8 | A | G | -0.032 | 0.002 | 210.249 |
| rs1260326 | 2 | C | T | 0.032 | 0.002 | 288.999 |
| rs12672217 | 7 | A | G | 0.014 | 0.002 | 48.302 |
| rs12702693 | 7 | T | C | 0.017 | 0.002 | 82.905 |
| rs12724708 | 1 | T | A | 0.024 | 0.002 | 147.622 |
| rs12773500 | 10 | T | C | 0.017 | 0.003 | 37.297 |
| rs12831751 | 12 | C | A | 0.017 | 0.002 | 67.084 |
| rs12907139 | 15 | A | G | -0.015 | 0.002 | 61.498 |
| rs1290786 | 3 | T | C | -0.014 | 0.002 | 56.645 |
| rs1291114 | 20 | C | G | 0.017 | 0.003 | 31.143 |
| rs12962050 | 18 | A | G | 0.015 | 0.002 | 58.522 |
| rs12997625 | 2 | T | C | -0.017 | 0.002 | 80.055 |
| rs13103161 | 4 | A | T | -0.028 | 0.002 | 223.423 |
| rs1325596 | 1 | A | G | 0.029 | 0.002 | 228.168 |
| rs1330826 | 9 | C | G | 0.016 | 0.002 | 49.610 |
| rs1340022 | 8 | C | T | 0.012 | 0.002 | 38.570 |
| rs1405227 | 1 | A | G | 0.013 | 0.002 | 41.602 |
| rs143384 | 20 | G | A | 0.073 | 0.002 | 1456.018 |
| rs1472852 | 4 | A | C | -0.064 | 0.003 | 602.133 |
| rs14976 | 2 | T | C | 0.014 | 0.002 | 51.840 |
| rs1514134 | 1 | C | T | -0.011 | 0.002 | 36.000 |
| rs1556659 | 10 | T | C | 0.016 | 0.002 | 66.422 |
| rs1584011 | 11 | G | T | 0.016 | 0.002 | 63.202 |
| rs17197114 | 14 | C | T | 0.018 | 0.003 | 50.126 |
| rs17205463 | 15 | T | C | -0.026 | 0.002 | 191.603 |
| rs17278379 | 1 | C | T | 0.023 | 0.003 | 60.732 |
| rs173135 | 17 | T | C | -0.034 | 0.003 | 129.201 |
| rs17400325 | 2 | C | T | 0.035 | 0.005 | 53.882 |
| rs17478946 | 12 | G | A | -0.019 | 0.002 | 83.591 |
| rs17681189 | 2 | A | C | -0.013 | 0.002 | 47.537 |
| rs17718736 | 3 | A | C | 0.012 | 0.002 | 33.062 |
| rs17773965 | 2 | T | C | -0.016 | 0.003 | 36.446 |
| rs17818592 | 16 | C | T | -0.013 | 0.002 | 46.097 |
| rs1786263 | 18 | T | G | -0.019 | 0.002 | 10<0.001 |
| rs1797070 | 1 | A | G | 0.022 | 0.002 | 108.755 |
| rs1933081 | 6 | A | T | 0.027 | 0.003 | 61.668 |
| rs2019203 | 17 | A | C | 0.019 | 0.002 | 98.950 |
| rs2025609 | 1 | G | C | 0.019 | 0.003 | 51.177 |
| rs2025808 | 1 | A | C | 0.012 | 0.002 | 30.752 |
| rs2035901 | 4 | G | A | 0.024 | 0.002 | 159.556 |
| rs212526 | 1 | C | T | 0.021 | 0.002 | 126.858 |
| rs2138374 | 2 | C | T | -0.015 | 0.002 | 55.502 |
| rs2140619 | 7 | G | A | 0.011 | 0.002 | 35.371 |
| rs2181834 | 10 | T | G | 0.025 | 0.002 | 178.714 |
| rs2212926 | 21 | A | C | -0.022 | 0.002 | 91.493 |
| rs2229840 | 12 | T | C | 0.034 | 0.003 | 172.013 |
| rs2230033 | 21 | A | G | -0.027 | 0.002 | 194.528 |
| rs2237485 | 7 | A | G | 0.019 | 0.002 | 68.962 |
| rs2240735 | 16 | T | C | 0.019 | 0.002 | 73.803 |
| rs2268718 | 6 | T | C | 0.014 | 0.002 | 45.081 |
| rs2274351 | 10 | T | C | 0.017 | 0.002 | 80.055 |
| rs2283200 | 11 | T | C | -0.028 | 0.004 | 44.762 |
| rs2289629 | 17 | A | G | -0.015 | 0.002 | 54.760 |
| rs2296316 | 14 | C | T | -0.019 | 0.002 | 102.116 |
| rs2303423 | 4 | C | T | 0.017 | 0.003 | 31.360 |
| rs2305141 | 2 | G | A | 0.018 | 0.002 | 92.767 |
| rs2347603 | 2 | A | T | -0.018 | 0.002 | 67.688 |
| rs2390669 | 2 | C | A | 0.017 | 0.003 | 38.617 |
| rs246177 | 16 | T | C | 0.021 | 0.002 | 114.489 |
| rs249677 | 5 | A | C | -0.011 | 0.002 | 29.702 |
| rs2521349 | 17 | A | G | 0.016 | 0.002 | 66.551 |
| rs2529090 | 7 | G | C | 0.014 | 0.003 | 29.593 |
| rs2578565 | 5 | T | C | -0.014 | 0.002 | 49.702 |
| rs2592208 | 17 | A | C | -0.012 | 0.002 | 42.593 |
| rs261223 | 5 | C | A | 0.018 | 0.002 | 84.833 |
| rs2648725 | 10 | A | T | 0.017 | 0.002 | 51.465 |
| rs2764264 | 6 | T | C | 0.020 | 0.002 | 93.444 |
| rs2788213 | 6 | A | G | 0.012 | 0.002 | 34.306 |
| rs2789365 | 1 | T | C | -0.015 | 0.002 | 58.241 |
| rs2807339 | 1 | C | T | 0.016 | 0.002 | 54.223 |
| rs2871865 | 15 | G | C | -0.049 | 0.003 | 270.053 |
| rs2871960 | 3 | C | A | 0.047 | 0.002 | 609.308 |
| rs291979 | 10 | A | G | 0.024 | 0.002 | 110.707 |
| rs2923411 | 8 | C | T | 0.013 | 0.002 | 44.678 |
| rs2925155 | 8 | T | C | -0.015 | 0.002 | 46.487 |
| rs293517 | 6 | C | T | -0.013 | 0.002 | 38.322 |
| rs2978362 | 18 | T | C | 0.011 | 0.002 | 31.125 |
| rs310796 | 12 | T | G | 0.014 | 0.002 | 50.410 |
| rs3116602 | 13 | G | T | -0.061 | 0.002 | 708.020 |
| rs3184504 | 12 | C | T | 0.018 | 0.002 | 92.767 |
| rs331917 | 5 | G | A | -0.013 | 0.002 | 44.678 |
| rs336630 | 3 | T | C | -0.011 | 0.002 | 31.125 |
| rs350832 | 19 | A | G | -0.017 | 0.002 | 51.465 |
| rs3764002 | 12 | T | C | 0.028 | 0.002 | 177.777 |
| rs3769598 | 2 | G | A | 0.017 | 0.003 | 40.111 |
| rs3778858 | 7 | T | G | 0.011 | 0.002 | 29.160 |
| rs3818416 | 13 | C | A | 0.028 | 0.002 | 160.828 |
| rs3822742 | 5 | A | C | 0.016 | 0.002 | 65.610 |
| rs395980 | 4 | G | T | -0.018 | 0.002 | 76.771 |
| rs4076108 | 3 | T | A | 0.017 | 0.002 | 62.553 |
| rs42039 | 7 | T | C | 0.048 | 0.002 | 478.016 |
| rs4282339 | 5 | A | G | -0.031 | 0.002 | 182.837 |
| rs4622329 | 12 | A | G | 0.015 | 0.002 | 55.502 |
| rs4640244 | 17 | G | A | -0.020 | 0.002 | 110.803 |
| rs4655345 | 1 | G | A | -0.025 | 0.002 | 167.634 |
| rs4752829 | 11 | A | G | 0.026 | 0.002 | 155.655 |
| rs4847378 | 1 | T | G | 0.014 | 0.002 | 51.235 |
| rs4852257 | 2 | G | T | -0.023 | 0.002 | 147.814 |
| rs4865956 | 5 | A | T | -0.026 | 0.002 | 150.938 |
| rs4870941 | 8 | C | G | -0.030 | 0.002 | 166.746 |
| rs488621 | 2 | G | A | 0.019 | 0.002 | 101.055 |
| rs4932439 | 15 | G | A | -0.015 | 0.003 | 36.481 |
| rs4940874 | 18 | G | A | 0.015 | 0.002 | 38.028 |
| rs496783 | 10 | G | A | -0.012 | 0.002 | 42.593 |
| rs568267 | 18 | T | C | 0.012 | 0.002 | 30.752 |
| rs5742915 | 15 | C | T | 0.025 | 0.002 | 170.370 |
| rs5753518 | 22 | A | G | 0.024 | 0.003 | 53.778 |
| rs577289 | 15 | T | A | -0.013 | 0.002 | 35.431 |
| rs591668 | 3 | A | G | -0.017 | 0.002 | 83.867 |
| rs604723 | 11 | C | T | -0.017 | 0.002 | 62.485 |
| rs6066122 | 20 | G | C | 0.013 | 0.002 | 30.489 |
| rs610694 | 12 | C | T | 0.014 | 0.002 | 51.235 |
| rs650508 | 2 | C | G | -0.013 | 0.002 | 42.250 |
| rs6543146 | 2 | G | T | 0.015 | 0.002 | 65.695 |
| rs6570509 | 6 | T | G | -0.024 | 0.002 | 135.002 |
| rs6593210 | 7 | A | G | 0.015 | 0.002 | 37.007 |
| rs6675858 | 1 | T | C | -0.014 | 0.002 | 35.480 |
| rs6693481 | 1 | C | T | -0.014 | 0.002 | 51.122 |
| rs670318 | 1 | C | T | 0.041 | 0.004 | 88.103 |
| rs6738207 | 2 | A | G | 0.013 | 0.002 | 44.678 |
| rs680882 | 7 | G | T | 0.013 | 0.002 | 36.547 |
| rs6849302 | 4 | G | A | 0.016 | 0.002 | 41.710 |
| rs6902109 | 6 | G | A | -0.017 | 0.002 | 77.255 |
| rs6931421 | 6 | G | T | -0.028 | 0.002 | 194.602 |
| rs700677 | 2 | A | C | 0.017 | 0.002 | 74.822 |
| rs704660 | 11 | T | C | 0.015 | 0.002 | 64.845 |
| rs713467 | 15 | A | G | 0.015 | 0.002 | 59.047 |
| rs7144307 | 14 | C | T | -0.012 | 0.002 | 37.210 |
| rs718603 | 6 | T | C | 0.013 | 0.002 | 38.914 |
| rs7220127 | 17 | C | T | -0.011 | 0.002 | 30.540 |
| rs7229520 | 18 | A | G | -0.022 | 0.002 | 125.439 |
| rs723149 | 7 | G | A | -0.028 | 0.002 | 211.013 |
| rs7328187 | 13 | G | T | 0.012 | 0.002 | 37.274 |
| rs7418410 | 1 | T | C | 0.016 | 0.002 | 66.551 |
| rs7485647 | 12 | A | G | -0.026 | 0.003 | 100.770 |
| rs7522400 | 1 | G | A | 0.013 | 0.002 | 34.382 |
| rs7598430 | 2 | T | C | -0.016 | 0.002 | 70.914 |
| rs7633464 | 3 | A | G | 0.018 | 0.002 | 84.833 |
| rs7679276 | 4 | G | A | -0.033 | 0.005 | 47.265 |
| rs7689420 | 4 | C | T | 0.047 | 0.003 | 347.448 |
| rs7731023 | 5 | G | A | 0.017 | 0.002 | 76.332 |
| rs7768973 | 6 | A | T | -0.024 | 0.002 | 159.556 |
| rs7816345 | 8 | T | C | 0.026 | 0.003 | 104.040 |
| rs7826059 | 8 | C | T | 0.011 | 0.002 | 32.490 |
| rs7828086 | 8 | C | T | 0.014 | 0.002 | 37.655 |
| rs7902 | 11 | G | A | 0.015 | 0.002 | 61.498 |
| rs798548 | 7 | C | T | -0.036 | 0.002 | 292.246 |
| rs8000973 | 13 | C | T | 0.013 | 0.002 | 49.739 |
| rs8018486 | 14 | G | A | -0.014 | 0.002 | 33.062 |
| rs8019890 | 14 | A | C | 0.025 | 0.002 | 173.129 |
| rs8020095 | 14 | A | G | -0.015 | 0.003 | 28.841 |
| rs8054549 | 16 | A | C | -0.025 | 0.002 | 174.517 |
| rs8084413 | 18 | A | G | -0.013 | 0.002 | 44.678 |
| rs867529 | 2 | C | G | 0.018 | 0.002 | 76.771 |
| rs876122 | 6 | G | A | 0.016 | 0.003 | 31.206 |
| rs900399 | 3 | G | A | 0.016 | 0.002 | 74.504 |
| rs905938 | 1 | C | T | 0.039 | 0.002 | 352.008 |
| rs909220 | 14 | A | G | -0.015 | 0.002 | 62.327 |
| rs9344126 | 6 | C | T | -0.019 | 0.002 | 94.806 |
| rs9388490 | 6 | T | C | 0.046 | 0.002 | 591.255 |
| rs947099 | 10 | A | G | 0.012 | 0.002 | 34.222 |
| rs951366 | 1 | C | T | 0.021 | 0.002 | 116.412 |
| rs963317 | 4 | G | A | -0.014 | 0.002 | 46.240 |
| rs9647379 | 3 | C | G | 0.022 | 0.002 | 128.047 |
| rs9828525 | 3 | T | C | 0.012 | 0.002 | 40.557 |
| rs9838614 | 3 | G | T | -0.019 | 0.002 | 94.806 |
| rs987666 | 7 | A | G | 0.019 | 0.003 | 40.695 |
| rs9905385 | 17 | G | A | -0.034 | 0.002 | 287.301 |
| rs9957318 | 18 | G | A | 0.019 | 0.002 | 87.422 |

**Supplemental Table S2.** Single nucleotide polymorphisms used as instrumental variables in the Mendelian randomization analyses of Hand grip strength (left).

| **SNP** | **Chr** | **EA** | **NEA** | **Beta** | **SE** | **F** |
| --- | --- | --- | --- | --- | --- | --- |
| rs10176878 | 2 | C | T | -0.013 | 0.002 | 46.625 |
| rs10205394 | 2 | C | G | -0.011 | 0.002 | 37.184 |
| rs10403906 | 19 | A | G | -0.010 | 0.001 | 45.539 |
| rs1044299 | 1 | T | C | 0.014 | 0.001 | 87.982 |
| rs10786706 | 10 | T | C | 0.010 | 0.001 | 45.265 |
| rs10788958 | 1 | G | C | 0.014 | 0.002 | 82.536 |
| rs10831903 | 11 | T | C | 0.009 | 0.002 | 37.746 |
| rs10988217 | 9 | G | A | -0.009 | 0.002 | 36.251 |
| rs11002322 | 10 | T | G | -0.010 | 0.002 | 40.351 |
| rs11003014 | 10 | G | A | 0.011 | 0.002 | 31.989 |
| rs11168357 | 12 | A | G | -0.010 | 0.002 | 31.099 |
| rs11243202 | 6 | C | T | 0.010 | 0.001 | 42.525 |
| rs11769549 | 7 | A | T | 0.020 | 0.003 | 43.367 |
| rs12473732 | 2 | T | C | 0.011 | 0.001 | 54.664 |
| rs12533765 | 7 | G | A | -0.009 | 0.002 | 31.014 |
| rs12673062 | 7 | A | G | -0.011 | 0.002 | 35.475 |
| rs13356200 | 5 | G | T | -0.009 | 0.002 | 32.899 |
| rs1434095 | 2 | C | T | 0.014 | 0.002 | 38.571 |
| rs1486925 | 8 | C | T | -0.010 | 0.002 | 42.736 |
| rs1556659 | 10 | T | C | 0.016 | 0.002 | 112.687 |
| rs16910750 | 9 | C | G | 0.011 | 0.002 | 30.369 |
| rs17282763 | 7 | C | T | 0.009 | 0.002 | 29.969 |
| rs17466480 | 15 | G | A | -0.012 | 0.002 | 59.928 |
| rs17630248 | 2 | C | T | -0.009 | 0.002 | 34.587 |
| rs2038760 | 6 | T | C | -0.012 | 0.002 | 33.662 |
| rs217181 | 16 | T | C | 0.012 | 0.002 | 40.378 |
| rs2359239 | 14 | T | C | -0.009 | 0.002 | 33.562 |
| rs2532111 | 17 | G | A | 0.010 | 0.002 | 43.584 |
| rs2789514 | 9 | A | G | 0.012 | 0.002 | 29.771 |
| rs2871865 | 15 | G | C | -0.022 | 0.002 | 88.538 |
| rs2871960 | 3 | C | A | 0.012 | 0.001 | 65.620 |
| rs2974438 | 5 | A | G | -0.010 | 0.002 | 30.505 |
| rs3819121 | 2 | C | T | 0.014 | 0.002 | 85.026 |
| rs4308051 | 18 | G | T | 0.016 | 0.002 | 77.333 |
| rs4335354 | 1 | A | C | -0.009 | 0.002 | 34.359 |
| rs4398863 | 8 | C | G | -0.009 | 0.002 | 31.471 |
| rs4498020 | 3 | A | C | -0.010 | 0.002 | 38.962 |
| rs4677601 | 3 | G | A | 0.009 | 0.001 | 37.213 |
| rs4737446 | 8 | T | G | 0.010 | 0.002 | 41.415 |
| rs6006984 | 22 | C | T | 0.010 | 0.002 | 35.453 |
| rs635538 | 18 | A | G | -0.022 | 0.003 | 66.361 |
| rs6802071 | 3 | T | C | -0.009 | 0.002 | 39.208 |
| rs6962338 | 7 | G | A | -0.021 | 0.004 | 35.034 |
| rs6977081 | 7 | T | G | 0.015 | 0.002 | 86.477 |
| rs7026798 | 9 | C | T | 0.008 | 0.002 | 29.891 |
| rs7124681 | 11 | A | C | -0.012 | 0.002 | 59.888 |
| rs7196917 | 16 | G | A | -0.012 | 0.002 | 61.100 |
| rs7197751 | 16 | T | G | -0.009 | 0.002 | 36.672 |
| rs7516571 | 1 | G | A | 0.009 | 0.002 | 30.661 |
| rs755547 | 17 | A | G | 0.017 | 0.002 | 75.744 |
| rs7571789 | 2 | C | T | 0.013 | 0.001 | 75.889 |
| rs7575451 | 2 | G | C | -0.010 | 0.002 | 39.237 |
| rs772014 | 10 | G | A | -0.011 | 0.002 | 48.866 |
| rs7856625 | 9 | T | C | -0.011 | 0.002 | 53.551 |
| rs7963801 | 12 | C | T | -0.010 | 0.002 | 47.866 |
| rs7970350 | 12 | T | C | -0.010 | 0.001 | 46.626 |
| rs823130 | 1 | T | C | -0.011 | 0.002 | 56.995 |
| rs9371201 | 6 | T | C | -0.009 | 0.002 | 35.209 |
| rs9371881 | 6 | A | G | 0.009 | 0.002 | 37.405 |
| rs9388769 | 6 | A | G | -0.014 | 0.002 | 79.371 |
| rs9866627 | 3 | A | C | -0.016 | 0.003 | 33.938 |
| rs9944324 | 16 | G | A | -0.009 | 0.002 | 32.437 |
| rs997850 | 4 | C | G | -0.009 | 0.002 | 33.727 |
| rs999493 | 17 | A | G | 0.013 | 0.002 | 69.914 |

**Supplemental Table S3.** Single nucleotide polymorphisms used as instrumental variables in the Mendelian randomization analyses of Hand grip strength (right).

| **SNP** | **Chr** | **EA** | **NEA** | **Beta** | **SE** | **F** |
| --- | --- | --- | --- | --- | --- | --- |
| rs10278546 | 7 | C | A | 0.011 | 0.002 | 32.591 |
| rs1043515 | 17 | G | A | 0.014 | 0.001 | 85.157 |
| rs1047891 | 2 | A | C | 0.010 | 0.002 | 36.814 |
| rs10483727 | 14 | C | T | -0.009 | 0.002 | 34.989 |
| rs10520770 | 18 | C | T | 0.012 | 0.001 | 61.463 |
| rs10770125 | 11 | G | A | 0.008 | 0.001 | 32.193 |
| rs10784502 | 12 | T | C | -0.011 | 0.001 | 56.039 |
| rs10798483 | 1 | A | G | 0.015 | 0.001 | 94.217 |
| rs10799428 | 1 | T | C | -0.014 | 0.002 | 56.892 |
| rs11022513 | 11 | T | C | -0.009 | 0.002 | 37.198 |
| rs11243202 | 6 | C | T | 0.012 | 0.001 | 60.757 |
| rs1125 | 6 | A | G | -0.010 | 0.002 | 40.572 |
| rs11998884 | 9 | T | C | 0.017 | 0.003 | 30.815 |
| rs12052508 | 2 | T | C | -0.013 | 0.002 | 34.295 |
| rs12412806 | 10 | A | G | -0.009 | 0.002 | 30.708 |
| rs12763284 | 10 | G | A | 0.010 | 0.001 | 41.529 |
| rs12823922 | 12 | G | A | -0.011 | 0.002 | 40.865 |
| rs12899474 | 15 | T | C | -0.015 | 0.002 | 38.532 |
| rs13355365 | 5 | T | C | -0.008 | 0.002 | 30.459 |
| rs13356200 | 5 | G | T | -0.009 | 0.002 | 36.191 |
| rs1440152 | 3 | G | C | 0.008 | 0.001 | 30.409 |
| rs1442883 | 2 | A | C | -0.011 | 0.002 | 38.371 |
| rs1486925 | 8 | C | T | -0.010 | 0.002 | 35.262 |
| rs1550115 | 2 | T | C | 0.015 | 0.002 | 79.626 |
| rs1556659 | 10 | T | C | 0.018 | 0.002 | 130.167 |
| rs1635527 | 12 | C | G | 0.010 | 0.001 | 42.561 |
| rs1892425 | 1 | A | G | 0.011 | 0.002 | 41.612 |
| rs2147461 | 1 | C | T | 0.013 | 0.002 | 33.788 |
| rs2194747 | 2 | G | A | 0.010 | 0.002 | 36.115 |
| rs2208562 | 9 | T | C | -0.012 | 0.002 | 57.669 |
| rs2244621 | 11 | T | C | 0.012 | 0.002 | 29.935 |
| rs2273555 | 10 | A | G | 0.011 | 0.002 | 52.566 |
| rs2296316 | 14 | C | T | -0.008 | 0.002 | 29.897 |
| rs2717351 | 7 | G | A | 0.013 | 0.002 | 48.440 |
| rs2854152 | 17 | G | A | 0.011 | 0.002 | 47.327 |
| rs2871865 | 15 | G | C | -0.024 | 0.002 | 104.216 |
| rs3118914 | 13 | T | G | -0.019 | 0.002 | 115.564 |
| rs3771498 | 2 | T | C | 0.014 | 0.001 | 90.495 |
| rs4369779 | 18 | C | T | 0.017 | 0.002 | 89.277 |
| rs4737446 | 8 | T | G | 0.010 | 0.002 | 40.099 |
| rs4784329 | 16 | C | A | -0.013 | 0.002 | 78.272 |
| rs4868110 | 5 | T | A | -0.010 | 0.002 | 37.309 |
| rs4927015 | 1 | A | G | 0.013 | 0.002 | 74.898 |
| rs6006984 | 22 | C | T | 0.010 | 0.002 | 38.646 |
| rs635538 | 18 | A | G | -0.022 | 0.003 | 68.228 |
| rs6473015 | 8 | C | A | 0.010 | 0.002 | 34.001 |
| rs6693567 | 1 | T | C | -0.010 | 0.002 | 33.752 |
| rs6693965 | 1 | T | G | -0.016 | 0.002 | 53.091 |
| rs6792762 | 3 | A | G | -0.009 | 0.002 | 36.189 |
| rs6962338 | 7 | G | A | -0.020 | 0.004 | 31.539 |
| rs6977081 | 7 | T | G | 0.013 | 0.002 | 65.526 |
| rs7034200 | 9 | A | C | 0.009 | 0.001 | 34.062 |
| rs7196917 | 16 | G | A | -0.011 | 0.002 | 49.578 |
| rs721101 | 6 | C | T | 0.009 | 0.002 | 31.975 |
| rs7301953 | 12 | A | G | -0.012 | 0.002 | 51.727 |
| rs7451021 | 6 | C | T | -0.016 | 0.002 | 96.780 |
| rs7549184 | 1 | A | G | 0.011 | 0.002 | 33.901 |
| rs7575451 | 2 | G | C | -0.011 | 0.002 | 46.466 |
| rs7576964 | 2 | T | G | 0.010 | 0.002 | 38.575 |
| rs7790322 | 7 | T | C | -0.009 | 0.002 | 32.494 |
| rs7963801 | 12 | C | T | -0.011 | 0.002 | 55.703 |
| rs823130 | 1 | T | C | -0.012 | 0.002 | 67.896 |
| rs852520 | 7 | A | C | -0.009 | 0.002 | 32.034 |
| rs911642 | 20 | T | C | 0.009 | 0.002 | 31.492 |
| rs935728 | 14 | T | C | 0.010 | 0.002 | 36.228 |
| rs9639938 | 7 | G | C | 0.009 | 0.001 | 34.111 |
| rs9652468 | 15 | A | G | -0.013 | 0.002 | 53.003 |
| rs9757079 | 3 | T | C | 0.010 | 0.002 | 37.674 |
| rs997850 | 4 | C | G | -0.009 | 0.002 | 34.766 |

**Supplemental Table S4.** Single nucleotide polymorphisms used as instrumental variables in the Mendelian randomization analyses of Walking pace.

| **SNP** | **Chr** | **EA** | **NEA** | **Beta** | **SE** | **F** |
| --- | --- | --- | --- | --- | --- | --- |
| rs10750025 | 11 | T | C | -0.008 | 0.001 | 37.453 |
| rs10828258 | 10 | G | A | -0.009 | 0.001 | 47.109 |
| rs10862220 | 12 | G | T | 0.008 | 0.001 | 38.757 |
| rs10883618 | 10 | A | G | 0.008 | 0.001 | 35.636 |
| rs11077815 | 17 | C | T | -0.007 | 0.001 | 29.792 |
| rs11848096 | 14 | C | T | -0.008 | 0.001 | 33.003 |
| rs12042959 | 1 | G | A | 0.013 | 0.002 | 48.058 |
| rs12461902 | 19 | A | G | -0.008 | 0.001 | 34.834 |
| rs13107325 | 4 | T | C | -0.024 | 0.002 | 101.506 |
| rs2037735 | 19 | T | C | -0.011 | 0.002 | 33.627 |
| rs205262 | 6 | G | A | -0.009 | 0.001 | 37.275 |
| rs2439823 | 10 | G | A | -0.007 | 0.001 | 32.616 |
| rs2602731 | 19 | G | A | -0.008 | 0.001 | 31.360 |
| rs2645979 | 12 | A | G | 0.009 | 0.001 | 42.609 |
| rs273512 | 19 | T | C | -0.010 | 0.001 | 56.084 |
| rs4516268 | 17 | A | C | 0.010 | 0.002 | 37.435 |
| rs4643373 | 17 | C | T | 0.008 | 0.001 | 31.247 |
| rs4715208 | 6 | G | A | -0.008 | 0.001 | 32.722 |
| rs4839898 | 6 | A | G | 0.013 | 0.002 | 40.156 |
| rs613872 | 18 | T | G | -0.015 | 0.002 | 77.512 |
| rs7789719 | 7 | C | T | 0.009 | 0.002 | 30.879 |
| rs7795394 | 7 | A | T | 0.009 | 0.001 | 50.555 |
| rs8010773 | 14 | C | T | -0.008 | 0.001 | 39.425 |
| rs8011870 | 14 | A | G | -0.008 | 0.001 | 30.977 |
| rs819167 | 20 | G | A | -0.016 | 0.003 | 37.130 |
| rs891387 | 18 | C | T | 0.008 | 0.001 | 38.539 |
| rs9783304 | 11 | T | G | 0.008 | 0.001 | 31.142 |

**Supplemental Table S5.** Instrumental variables of Barrett's esophagus

| SNP | Chr | EA | NEA | Beta | SE | F |
| --- | --- | --- | --- | --- | --- | --- |
| rs10039754 | 5 | G | A | 0.0837988 | 0.0150523 | 30.99234768 |
| rs10104032 | 8 | A | C | 0.0925762 | 0.0155502 | 35.4414392 |
| rs10207635 | 2 | T | A | 0.136706 | 0.0220023 | 38.60322564 |
| rs10982622 | 9 | G | A | -0.0854904 | 0.0151732 | 31.74424049 |
| rs11792928 | 9 | T | C | 0.0976628 | 0.016616 | 34.54538068 |
| rs1247942 | 12 | C | G | -0.0961235 | 0.0153517 | 39.20403866 |
| rs13195040 | 6 | G | A | -0.162354 | 0.0245982 | 43.56161141 |
| rs1868915 | 2 | A | C | 0.0892647 | 0.0153358 | 33.87905083 |
| rs2597301 | 3 | G | C | -0.113971 | 0.0161887 | 49.56206293 |
| rs2861695 | 2 | G | A | -0.107195 | 0.0188427 | 32.36289745 |
| rs3072 | 2 | C | T | 0.110363 | 0.015734 | 49.19866408 |
| rs622217 | 6 | C | T | -0.0911286 | 0.0150412 | 36.70532144 |
| rs7187365 | 16 | C | T | -0.111392 | 0.019545 | 32.48039191 |
| rs739414 | 16 | T | C | -0.101127 | 0.0176328 | 32.89094046 |
| rs7720419 | 5 | T | A | 0.0904389 | 0.0155265 | 33.92719967 |
| rs8102046 | 19 | G | T | -0.0917291 | 0.0151203 | 36.80256225 |

**Supplemental Table S6.** The result of the direct causal effect of physical performance or parameters of sarcopenia on BE by adjusting BMI, smoking traits, alcohol intake, protein intake, lipid intake, carbohydrate intake, sugar intake, as well as vitamin D and calcium.

|  | **ID.exposure** | **Exposure** | ***P* value** | **OR** | **OR_lci95** | **OR_uci95** |
| --- | --- | --- | --- | --- | --- | --- |
| Appendicular lean mass | ieu-b-4815 | Body mass index | 0.094 | 1.071 | 0.988 | 1.159 |
|  | ebi-a-GCST90000025 | Appendicular lean mass adjusted by Body mass index | <0.001 | 0.618 | 0.534 | 0.716 |
|  | ebi-a-GCST009967 | Smoking | 0.510 | 1.110 | 0.811 | 0.949 |
|  | ebi-a-GCST90000025 | Appendicular lean mass adjusted by Smoking | <0.001 | 0.755 | 0.570 | 0.656 |
|  | ieu-b-73 | Alcohol | 0.358 | 1.452 | 0.655 | 3.218 |
|  | ebi-a-GCST90000025 | Appendicular lean mass adjusted by Alcohol | <0.001 | 0.610 | 0.524 | 0.709 |
|  | Carbohydrate intake | Carbohydrate intake | 0.814 | 1.097 | 0.507 | 2.373 |
|  | ebi-a-GCST90000025 | Appendicular lean mass adjusted by Carbohydrate intake | <0.001 | 0.628 | 0.545 | 0.723 |
|  | Fat intake | Fat intake | 0.455 | 1.371 | 0.599 | 3.138 |
|  | ebi-a-GCST90000025 | Appendicular lean mass adjusted Fat intake | <0.001 | 0.628 | 0.545 | 0.723 |
|  | Sugar intake | Sugar intake | 0.939 | 0.974 | 0.489 | 1.938 |
|  | ebi-a-GCST90000025 | Appendicular lean mass adjusted by Sugar intake | <0.001 | 0.625 | 0.542 | 0.720 |
|  | Protein intake | Protein intake | 0.741 | 0.884 | 0.424 | 1.842 |
|  | ebi-a-GCST90000025 | Appendicular lean mass adjusted by Protein intake | <0.001 | 0.628 | 0.545 | 0.723 |
|  | ebi-a-GCST005367 | Vitamin D levels | 0.358 | 1.692 | 0.827 | 1.183 |
|  | ebi-a-GCST90000025 | Appendicular lean mass adjusted by Vitamin D levels | <0.001 | 0.738 | 0.557 | 0.641 |
|  | ebi-a-GCST90025990 | Calcium levels | 0.079 | 1.256 | 0.974 | 1.620 |
|  | ebi-a-GCST90000025 | Appendicular lean mass adjusted by Calcium levels | <0.001 | 0.640 | 0.555 | 0.738 |
| Hand grip strength (left) | ieu-b-4815 | Body mass index | 0.004 | 1.107 | 1.032 | 1.187 |
|  | ukb-b-7478 | Hand grip strength (left) adjusted by Body mass index | <0.001 | 0.432 | 0.280 | 0.666 |
|  | ebi-a-GCST009967 | Smoking | 0.812 | 1.493 | 0.731 | 1.044 |
|  | ukb-b-7478 | Hand grip strength (left) adjusted by Smoking | 0.006 | 0.792 | 0.251 | 0.446 |
|  | ieu-b-73 | Alcohol | 0.113 | 0.509 | 0.221 | 1.174 |
|  | ukb-b-7478 | Hand grip strength (left) adjusted by Alcohol | 0.045 | 0.558 | 0.316 | 0.986 |
|  | Carbohydrate intake | Carbohydrate intake | 0.114 | 2.224 | 0.826 | 5.986 |
|  | ukb-b-7478 | Hand grip strength (left) adjusted by Carbohydrate intake | 0.006 | 0.473 | 0.277 | 0.807 |
|  | Fat intake | Fat intake | 0.369 | 0.392 | 0.051 | 3.029 |
|  | ukb-b-7478 | Hand grip strength (left) adjusted by Fat intake | 0.019 | 0.506 | 0.286 | 0.893 |
|  | Sugar intake | Sugar intake | 0.027 | 3.298 | 1.146 | 9.494 |
|  | ukb-b-7478 | Hand grip strength (left) adjusted by Sugar intake | 0.009 | 0.501 | 0.298 | 0.842 |
|  | Protein intake | Protein intake | 0.312 | 1.639 | 0.629 | 4.271 |
|  | ukb-b-7478 | Hand grip strength (left) adjusted by Protein intake | 0.005 | 0.465 | 0.273 | 0.790 |
|  | ebi-a-GCST005367 | Vitamin D levels | 0.408 | 1.963 | 0.760 | 1.222 |
|  | ukb-b-7478 | Hand grip strength (left) adjusted by Vitamin D levels | 0.003 | 0.753 | 0.252 | 0.436 |
|  | ebi-a-GCST90025990 | Calcium levels | 0.544 | 0.951 | 0.808 | 1.119 |
|  | ukb-b-7478 | Hand grip strength (left) adjusted by Calcium levels | 0.013 | 0.307 | 0.121 | 0.781 |
| Hand grip strength (right) | ieu-b-4815 | Body mass index | 0.203 | 1.073 | 0.963 | 1.195 |
|  | ukb-b-10215 | Hand grip strength (right) adjusted by Body mass index | 0.001 | 0.402 | 0.230 | 0.702 |
|  | ebi-a-GCST009967 | Smoking | 0.707 | 1.495 | 0.762 | 1.067 |
|  | ukb-b-10215 | Hand grip strength (right) adjusted by Smoking | 0.001 | 0.723 | 0.255 | 0.429 |
|  | ieu-b-73 | Alcohol | 0.387 | 0.689 | 0.295 | 1.604 |
|  | ukb-b-10215 | Hand grip strength (right) adjusted by Alcohol | 0.009 | 0.487 | 0.284 | 0.835 |
|  | Carbohydrate intake | Carbohydrate intake | 0.277 | 1.725 | 0.645 | 4.614 |
|  | ukb-b-10215 | Hand grip strength (right) adjusted by Carbohydrate intake | 0.002 | 0.445 | 0.266 | 0.744 |
|  | Fat intake | Fat intake | 0.221 | 0.333 | 0.057 | 1.938 |
|  | ukb-b-10215 | Hand grip strength (right) adjusted by Fat intake | 0.006 | 0.474 | 0.277 | 0.809 |
|  | Sugar intake | Sugar intake | 0.239 | 1.920 | 0.648 | 5.683 |
|  | ukb-b-10215 | Hand grip strength (right) adjusted by Sugar intake | 0.002 | 0.450 | 0.269 | 0.753 |
|  | Protein intake | Protein intake | 0.251 | 1.818 | 0.655 | 5.045 |
|  | ukb-b-10215 | Hand grip strength (right) adjusted by Protein intake | 0.002 | 0.439 | 0.263 | 0.732 |
|  | ebi-a-GCST005367 | Vitamin D levels | 0.450 | 1.911 | 0.750 | 1.197 |
|  | ukb-b-10215 | Hand grip strength (right) adjusted by Vitamin D levels | 0.001 | 0.702 | 0.253 | 0.421 |
|  | ebi-a-GCST90025990 | Calcium levels | 0.553 | 0.952 | 0.809 | 1.120 |
|  | ukb-b-10215 | Hand grip strength (right) adjusted by Calcium levels | 0.003 | 0.321 | 0.152 | 0.678 |
| Walking pace | ieu-b-4815 | Body mass index | 0.591 | 1.023 | 0.942 | 1.110 |
|  | ukb-b-4711 | Walking pace adjusted by Body mass index | <0.001 | 0.038 | 0.015 | 0.098 |
|  | ebi-a-GCST009967 | Smoking | 0.451 | 2.154 | 0.711 | 1.238 |
|  | ukb-b-4711 | Walking pace adjusted by Smoking | <0.001 | 0.099 | 0.013 | 0.036 |
|  | ieu-b-73 | Alcohol | 0.838 | 1.092 | 0.469 | 2.542 |
|  | ukb-b-4711 | Walking pace adjusted by Alcohol | <0.001 | 0.036 | 0.014 | 0.092 |
|  | Carbohydrate intake | Carbohydrate intake | 0.515 | 1.314 | 0.577 | 2.994 |
|  | ukb-b-4711 | Walking pace adjusted by Carbohydrate intake | <0.001 | 0.040 | 0.017 | 0.094 |
|  | Fat intake | Fat intake | 0.484 | 0.451 | 0.048 | 4.197 |
|  | ukb-b-4711 | Walking pace adjusted by Fat intake | <0.001 | 0.042 | 0.017 | 0.102 |
|  | Sugar intake | Sugar intake | 0.149 | 1.855 | 0.802 | 4.290 |
|  | ukb-b-4711 | Walking pace adjusted by Sugar intake | <0.001 | 0.044 | 0.019 | 0.098 |
|  | Protein intake | Protein intake | 0.485 | 0.726 | 0.295 | 1.783 |
|  | ukb-b-4711 | Walking pace adjusted by Protein intake | <0.001 | 0.038 | 0.015 | 0.096 |
|  | ebi-a-GCST005367 | Vitamin D levels | 0.398 | 1.784 | 0.794 | 1.190 |
|  | ukb-b-4711 | Walking pace adjusted by Vitamin D levels | <0.001 | 0.086 | 0.013 | 0.034 |
|  | ebi-a-GCST90025990 | Calcium levels | 0.534 | 0.955 | 0.828 | 1.103 |
|  | ukb-b-4711 | Walking pace adjusted by Calcium levels | <0.001 | 0.032 | 0.008 | 0.118 |

The reported values were calculated by the random effects IVW method.  *OR, odds ratio; CI, confidence interval; IVW, inverse variance weighted method; BE, Barrett's esophagus.*

**Supplemental Table S7.** The result of heterogeneity and pleiotropy test of BE and risk of sarcopenia in reverse MR analyses

| **Outcome** | **Heterogeneity** | | **Pleiotropy** | |
| --- | --- | --- | --- | --- |
|  | **Q** | ***P* value** | **Egger intercept** | ***P* value** |
| Appendicular lean mass | 12.0061 | 0.1509 | 0.0062 | 0.2281 |
| Hand grip strength (left) | 28.2897 | 0.0198 | -0.0053 | 0.1352 |
| Hand grip strength (right) | 48.0250 | 2.52E-05 | -0.0040 | 0.3980 |
| Walking pace | 46.6034 | 4.26E-05 | -0.0021 | 0.5988 |

*BE, Barrett's esophagus.*

**Supplemental Table S8.** The result of the F statistic of sarcopenia and risk of BE in multivariable MR analyses.

| **Comfounders** | **Appendicular lean mass** | | **Hand grip strength (left)** | | **Hand grip strength (right)** | | **Walking pace** | |
| --- | --- | --- | --- | --- | --- | --- | --- | --- |
| **adjusted by BMI** | FBMI=1.618 | FAlm=105.065 | FBMI=4.947 | Fleft=42.424 | FBMI=3.849 | Fright=50.363 | FBMI=7.298 | FWp=13.338 |
| **adjusted by smoking** | F_smo_=1.075 | F_Alm_=91.451 | F_smo_=0.982 | F_left_=23.082 | F_smo_=1.014 | F_right_=44.381 | F_smo_=0.795 | F_Wp_=10.267 |
| **adjusted by alcohol** | Falc=3.485 | FAlm=102.983 | Falc=11.526 | Fleft=40.663 | Falc=10.785 | Fright=43.155 | Falc=17.923 | FWp=30.504 |
| **adjusted by carbohydrate intake** | FCHO=1.626 | FAlm=102.973 | FCHO=3.890 | Fleft=25.041 | FCHO=3.902 | Fright=30.131 | FCHO=7.646 | FWp=27.154 |
| **adjusted by fat intake** | Ffat=1.415 | FAlm=87.731 | Ffat=0.995 | Fleft=6.959 | Ffat=1.336 | Fright=17.575 | Ffat=1.289 | FWp=10.466 |
| **adjusted by sugar intake** | Fsug=1.770 | FAlm=74.522 | Fsug=2.933 | Fleft=26.942 | Fsug=2.911 | Fright=29.463 | Fsug=6.354 | FWp=29.873 |
| **adjusted by protein intake** | Fpro=1.787 | FAlm=98.556 | Fpro=4.279 | Fleft=47.929 | Fpro=3.753 | Fright=50.358 | Fpro=7.638 | FWp=31.503 |
| **adjusted by Vitamin D levels** | F_VD_=10.020 | F_Alm_=101.296 | F_VD_=32.862 | F_left_=45.638 | F_VD_=31.234 | F_right_=48.590 | F_VD_=68.471 | F_Wp_=32.068 |
| **adjusted by calcium levels** | F_cal_=112.894 | F_Alm_=75.453 | F_cal_=86.462 | F_left_=8.028 | F_cal_=94.335 | F_right_=11.621 | F_cal_=92.070 | F_Wp_=4.192 |

*BE, Barrett's esophagus; BMI, Body mass index*
